# Exploration of Anti-HIV Phytocompounds against SARS-CoV-2 Main Protease: Structure-Based Screening, Molecular Simulation, ADME Analysis and Conceptual DFT Studies

**DOI:** 10.3390/molecules27238288

**Published:** 2022-11-28

**Authors:** Mahadevamurthy Murali, Hittanahallikoppal Gajendramurthy Gowtham, Natarajamurthy Shilpa, Hemanth Kumar Naguvanahalli Krishnappa, Ana E. Ledesma, Anisha S. Jain, Ali A. Shati, Mohammad Y. Alfaifi, Serag Eldin I. Elbehairi, Raghu Ram Achar, Ekaterina Silina, Victor Stupin, Joaquín Ortega-Castro, Juan Frau, Norma Flores-Holguín, Kestur Nagaraj Amruthesh, Chandan Shivamallu, Shiva Prasad Kollur, Daniel Glossman-Mitnik

**Affiliations:** 1 Department of Studies in Botany, University of Mysore, Mysore 570006, India; 2 Department of PG Studies in Biotechnology, Nrupathunga University, Nrupathunga Road, Bangalore 560001, India; 3 Department of Studies in Microbiology, University of Mysore, Mysore 570006, India; 4 Post Graduate Department of Botany, Maharani’s Science College for Women, JLB Road, Mysuru 570005, India; 5 Centro de InvestigaciónenBiofísicaAplicada y Alimentos, Facultad de Ciencias Exactas y Tecnologías (FCEyN), Universidad Nacional de Santiago del Estero (CIBAAL-UNSE-CONICET), Santiago del Estero 4206, Argentina; 6 Department of Microbiology, JSS Academy of Higher Education and Research, Mysuru 570015, India; 7 Biology Department, Faculty of Science, King Khalid University, Abha 9004, Saudi Arabia; 8 Cell Culture Lab, Egyptian Organization for Biological Products and Vaccines (VACSERA Holding Company), 51 Wezaret El-Zeraa St., Giza 12511, Egypt; 9 Division of Biochemistry, School of Life Sciences, JSS Academy of Higher Education and Research, Mysuru 570015, India; 10 Department of Hospital Surgery, N.I. Pirogov Russian National Research Medical University, Moscow 117997, Russia; 11 Departament de Química, Facultat de Ciences, Universitat de les IllesBalears, E-07122 Palma de Malllorca, Spain; 12 Laboratorio Virtual NANOCOSMOS, Departamento de Medio Ambiente y Energía, Centro de Investigaciónen Materiales Avanzados, Chihuahua 31136, Mexico; 13 Department of Biotechnology and Bioinformatics, JSS Academy of Higher Education and Research, Mysuru 570015, India; 14 School of Physical Sciences, Amrita Vishwa Vidyapeetham, Mysuru Campus, Mysuru 570026, India

**Keywords:** SARS-CoV-2, main protease, anti-HIV, bioactive compounds, conceptual DFT

## Abstract

The ever-expanding pandemic severe acute respiratory syndrome coronavirus 2 (SARS-CoV-2) infection has gained attention as COVID-19 and caused an emergency in public health to an unmatched level to date. However, the treatments used are the only options; currently, no effective and licensed medications are available to combat disease transmission, necessitating further research. In the present study, an in silico-based virtual screening of anti-HIV bioactive compounds from medicinal plants was carried out through molecular docking against the main protease (M^pro^) (PDB: 6LU7) of SARS-CoV-2, which is a key enzyme responsible for virus replication. A total of 16 anti-HIV compounds were found to have a binding affinity greater than −8.9 kcal/mol out of 150 compounds screened. Pseudohypericin had a high affinity with the energy of −10.2 kcal/mol, demonstrating amino acid residual interactions with LEU141, GLU166, ARG188, and GLN192, followed by Hypericin (−10.1 kcal/mol). Moreover, the ADME (Absorption, Distribution, Metabolism and Excretion) analysis of Pseudohypericin and Hypericin recorded a low bioavailability (BA) score of 0.17 and violated Lipinski’s rule of drug-likeness. The docking and molecular simulations indicated that the quinone compound, Pseudohypericin, could be tested in vitro and in vivo as potent molecules against COVID-19 disease prior to clinical trials.This was also supported by the theoretical and computational studies conducted. The global and local descriptors, which are the underpinnings of Conceptual Density FunctionalTheory (CDFT) have beenpredicted through successful model chemistry, hoping that they could be of help in the comprehension of the chemical reactivity properties of the molecular systems considered in this study.

## 1. Introduction

New diseases have emerged from the beginning of the 21st century, viz., SARS-CoV-2, Middle east respiratory syndrome coronavirus (MERS-CoV), H1N1 swine influenza, Ebola virus, Zika virus, and Nipah virus, etc., which are life-threatening to humankind [1,2]. The researchers were able to stop these diseases from spreading over the world or found successful drugs to inhibit them. Even though several safe and effective COVID-19 vaccines are being used to break viral spread and infection, COVID-19 safety guidelines must be followed worldwide [3]. In addition, there are currently few vaccine safety assessment data available on immunization in pregnant women and infants (WHO). Currently, the studies are looking at using available medications for other diseases, as the repurposing of the same has cured/inhibited the spread of other viruses, such as Zika and Hepatitis C, along with Ebola [4,5]. The clinical studies on the use of Lopinavir–Ritonavir, which is already in use, have given a ray of hope for their usage against COVID-19 [6]. 

The coronavirus outbreak caused by severe acute respiratory syndrome coronavirus (SARS-CoV-2), also known as COVID-19, belongs to the beta coronavirus family [7]. The pathogen has halted human activity to its maximum and severely damaged the lifestyle of humans, apart from breaking the backbone of the economy throughout the world. COVID-19 is highly contagious and mainly damages the infected person’s respiratory system, resulting in higher oxidation and inflammation in the respiratory tract, necessitating specific treatment [8,9]. Antiviral and corticosteroid medicines are currently used to treat the infection, along with mechanical respiratory assistance, which has not substantially impacted the people who have been infected severely [10]. Internalization of SARS-CoV-2 into the human cells results in the development of an RNA template that directs the translation of two polyproteins (pp1a and pp1ab), which encode many vital non-structural proteins (NSPs), including main protease (M^pro^)-NSPs [11,12]. The M^pro^ NSPs contain both the polyproteins in a sequence-specific style which acts at eleven proteolytic cleavage sites out of sixteen sites, including the cleavage site (Leu-Gln*Ser-Ala-Gly) to generate critical NSPs that have a significant impact on the pathogen infection by the formation of a replication-transcription complex [13,14]. The cleavage site in M^pro^-NSPs has invited better attention from other NSPs because of its crucial and conserved role in the proteolysis of viral replicase polyproteins [15,16,17,18].

Molecular docking is a bioinformatic modelling tool that is used to predict the interactions of a protein (enzyme) with ligand molecules; several reports [19,20,21] are available for the screening of various compounds before employing them in biological studies or to predict the mode of inhibition offered during the in vitro/in vivo studies, and Food and Drug Administration (FDA) approved antiviral drugs [22,23] as the inhibitor of SARS-CoV-2 M^pro^. It may be well noted that even though there are more than four vaccines that have been approved by the World Health Organization (WHO) in the recent past, the virus has mutated very fast, and a new variant called Delta is posing a threat all around the world and is declared as a variant of concern by WHO. It has been observed that the recent wave in India is directly co-related to these variants, (B.1.617.2 strain) and now it is a concern in the United Kingdom and the United States of America. The researchers are now trying to validate the efficacy of the approved vaccines against this Delta variant. However, they have not conclusively stated its efficacy in controlling this variant of global concern. The bioactive compounds with anti-HIV properties from plant sources may facilitate identifying compounds with inhibitory potential against SARS-CoV-2. In our previous studies, the selected anti-HIV bioactive compounds were evaluated against SARS-CoV-2 RNA-dependent RNA polymerase (RdRp) and the results of the molecular docking and ADME and Toxicity studies proved to be effective against SARS-CoV-2 [24]. Hence, a systematic study on in silico-based drug repurposing methods using molecular docking and molecular simulation studies was performed against M^pro^ of SARS-CoV-2 by utilizing the available anti-HIV bioactive compounds from plantsbased on the literature [25,26].Therefore, the present study will help to identify other potential drugs against SARS-CoV-2 [25,26,27,28,29,30]. Additionally, the global and local descriptors that are the foundations of Conceptual Density Functional Theory (CDFT) have been predictedthroughsuccessfulmodel chemistry in the hopes that they will aid in understanding the chemical reactivity properties of the studied molecular systems.

## 2. Results

### 2.1. Molecular Docking Analysis

**Figure 1:**
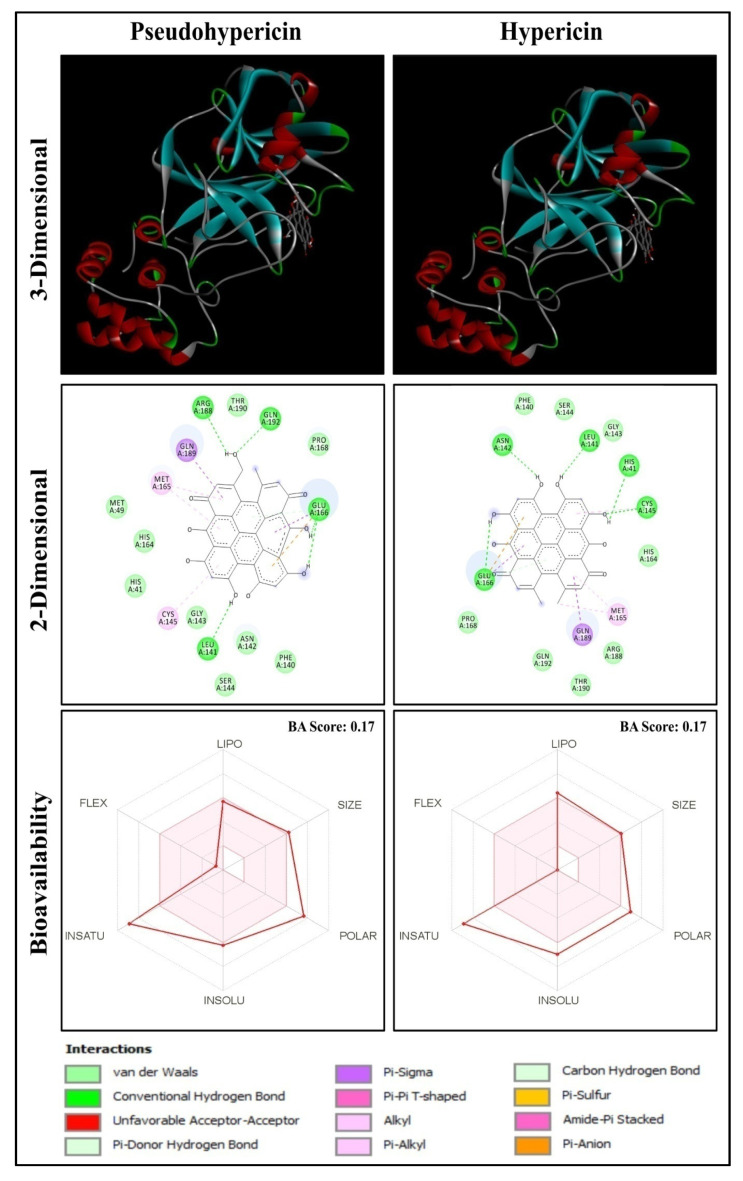
Anti-HIV compounds Pseudohypericin and Hypericin docked with M^pro^ of SARS-CoV-2.

**Figure 2:**
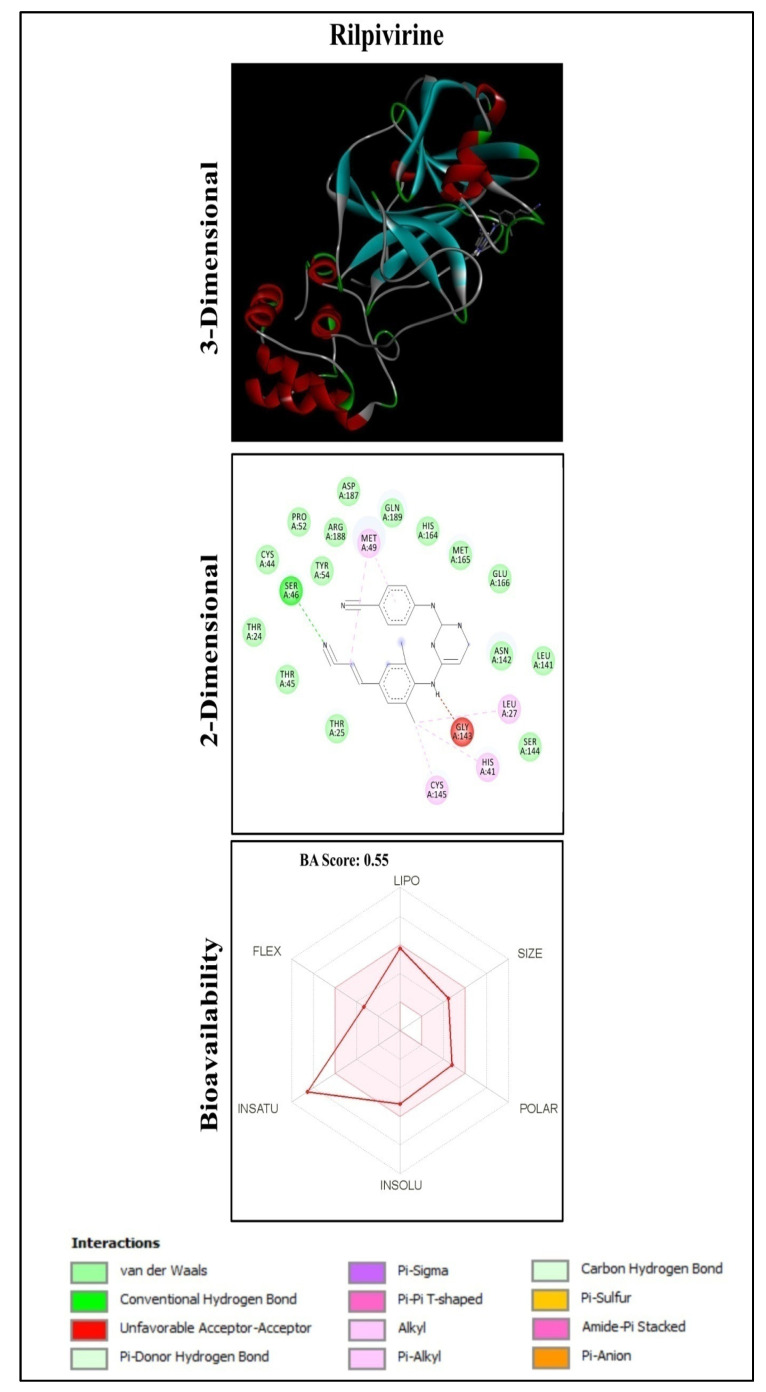
Rilpivirine docked with M^pro^ of SARS-CoV-2.

**Figure 3:**
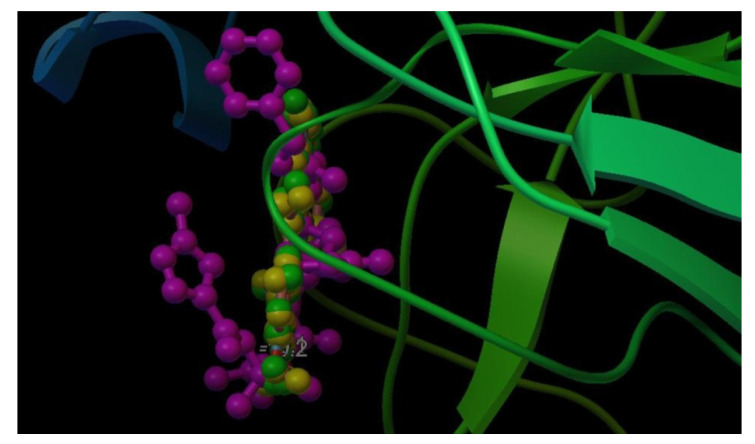
Interaction of N3 inhibitor (magenta), Pseudohypericin (green), and Hypericin (yellow).

A total of 150 anti-HIV bioactive compounds from medicinal plants and 18 anti-HIV drugs were docked against the target COVID-19 main protease and ranked based on their docking score. The corresponding canonical SMILES are displayed in Supplementary Table S1. Among the compounds subjected to virtual screening through molecular docking, 16 offered less than −8.9 kcal/mol docking score, representing the best-bound ligand conformations (Table 1, Figure 1 and Figures S1–S7). The molecular docking analysis showed that quinone compounds, Pseudohypericin and Hypericin, exhibited the lowest binding energy (BE) (−10.2 and −10.1 kcal/mol, respectively), followed by the flavonoid type of compound, Robustaflavone, with BE of −9.7 kcal/mol. Among the drugs used, Rilpivirine exhibited the lowest binding energy of −8.9 kcal/mol (Figure 2 and Supplementary Table S2), forming only a single hydrogen bond with SER46.Pseudohypericin interacted with the binding site residues (viz., LEU141, GLU166, ARG188 and GLN192) of M^pro^ of SARS-CoV-2 by forming four hydrogen bonds. Similarly, hydrogen bonds with the LEU141 and GLU166 residues were also noticed with Hypericin along with HIS41, ASN142 and CYS145 residues. Along with the H bonds, van der Waals, carbon-hydrogen, and pi-anion bonds, the binding site residues of the SARS-CoV-2 main protease were observed in Pseudohypericin and Hypericin. The interaction studies through molecular docking analysis and functional group analysis revealed that both Pseudohypericinand Hypericin bound to the same pocket of the binding site, as noticed (Figure 3).

### 2.2. Functional Group Analysis

The remaining molecules, including flavonoids, phenolic and alkaloid compounds, exhibited lower binding energies than the before analyzed molecules, with a range of binding energy from −9.7 to −8.9 kcal/mol, which was better thanthe inhibitor N3 molecule (−7.9 kcal/mol). The anthraquinone structures of Pseudohypericin and Hypericin with the fused aromatic ring are many hydroxyl (OH) groups, representing a high electronegative source, which contributes to the hydrogen bonding interaction with the binding site of M^pro^ protein. On the other hand, flavonoids molecules such as Robustaflavone, procyanidin B2 and Agathisflavone, without fused rings but OH groups and 2-phenyl-chromone nucleus, slightly reduce the interaction with receptors and decrease their binding affinity. Similar behavior can be observed for the phenolic compound with hydroxyl groups (–OH), such as (-)-Epicatechin-(4beta→8)-(-)-epigallocatechin and (-)-Epicatechin(4.beta.→8)(-)-4′-methylepigallocatechin.

### 2.3. Molecular Dynamics Simulation

**Figure 4:**
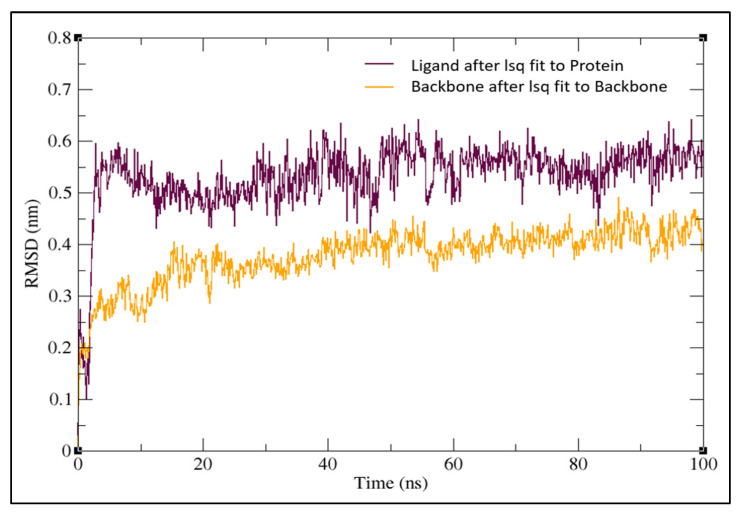
Molecular dynamics root mean square deviation (RMSD) plot for the docked complex of Pseudohypericin with COVID-19 main protease.

**Figure 5:**
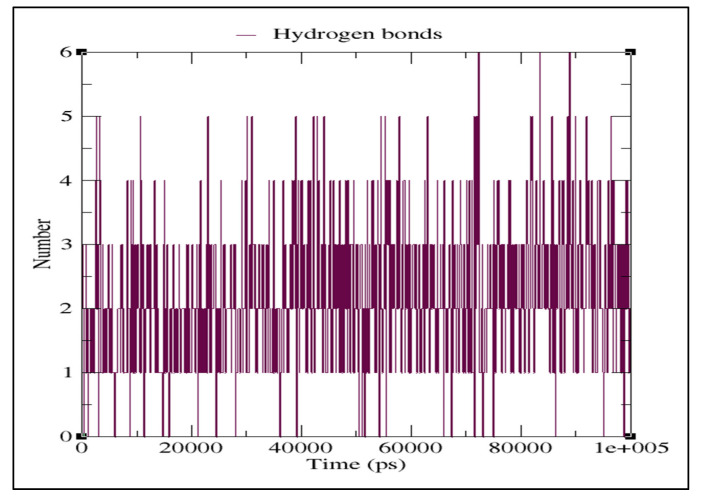
Hydrogen bonds (HBonds) plot for the docked complex of Pseudohypericin with COVID-19 main protease.

**Figure 6:**
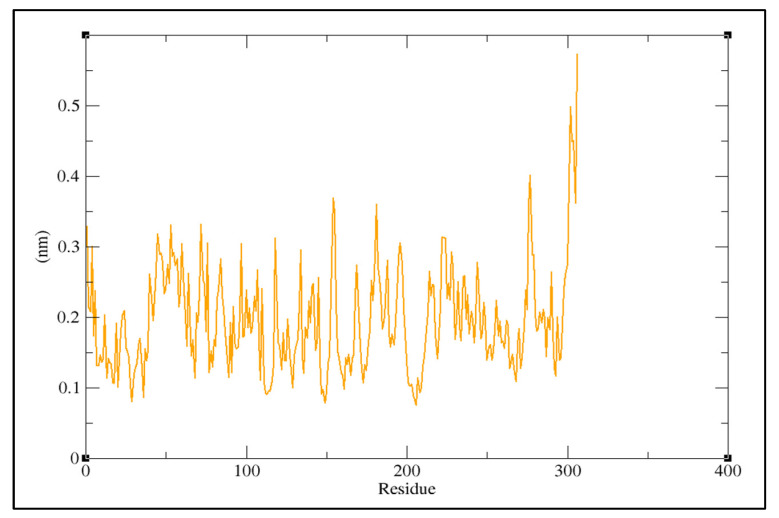
Molecular dynamics root mean square fluctuation (RMSF) of the protein backbone.

TheMD simulation was carried out with the docked complex of Pseudohypericin with M^pro^ protein as it showed a lower binding energy value when compared to Hypericin to characterize the variations of residues at 100 ns. The simulation results were compared to those obtained with the co-crystalized peptide inhibitor (N3). The lower RMSD value indicated the greater stability of the protein. In the present study, the Pseudohypericincompound reached a maximum RMSD value of 0.62 nm and fluctuated between 0.5–0.6 nm from 5 ns to 100 ns. Initially, the graph peaked from 0.28 nm to 0.6 nm in a 5 ns time run (Figure 4). The hydrogen bonds between Pseudohypericin and COVID-19 main protease throughout the simulation are shown in Figure 5. A maximum of six H-bonds was maintained, with an average of three throughout the simulation time and the Root Mean Square Fluctuations (RMSFs) were studied (Figure 6). The C terminal residues of the protein displayed substantial variations, with RMSF peaking at 0.6 nm and fluctuating between 0.1 to 0.3 nm.

### 2.4. ADME Properties of Ligands

The results of the ADME test showed the lipophilicity, pharmacokinetics, drug-likeness, and medicinal chemistry friendliness of the selected potential compounds (Tables S3–S5). The lipophilicity of Pseudohypericin was iLOGP (2.94), XLOGP3 (4.46), WLOGP (4.73), MLOGP (0.58), SILICOS-IT (4.77) and Consensus Log Po/w of 3.50, while Hypericin with iLOGP (3.10), XLOGP3 (5.71), WLOGP (5.76), MLOGP (1.36), SILICOS-IT (5.37) and Consensus Log Po/w of 4.26. Pharmacokinetics data predicted that both Pseudohypericin and Hypericin were of low Gastrointestinal (GI) absorption and were not blood-brain barrier (BBB) permeants. They do not act as P-glycoprotein (P-gp) substrates and do not inhibit CYP1A2, CYP2D6 and CYP3A4 cytochromes, except CYP2C19 and CYP2C9. Skin permeation kinetics (Log Kp) was found to be −6.31 cm/s and −5.32 cm/s for Pseudohypericin and Hypericin, respectively. These two compounds recorded a low bioavailability (BA) score of 0.17 and violated Lipinski’s drug-likeness rule. Medicinal chemistry properties for these compounds were found to violate the Pan Assay Interference Structures (PAINS) laws with an alert of one D, Brenk’s laws with two alerts of being polycyclic aromatic hydrocarbon two and three, and no Lead likeness with molecular weight (MW) of greater than 350 and XLOGP3 of greater than 3.5. The synthetic accessibility score was 3.95 and 3.89 for Pseudohypericin and Hypericin, respectively.

In Table S3, the selected biomolecules under study have been labelled following the numbers presented in Table 1. At the same time, GI means Gastrointestinal, BBB stands for Blood-Brain-Barrier, P-gp corresponds to P-glycoprotein and CYP is acronymous for Cytochrome P450.In Table S4, the selected biomolecules under study have been labelled following the numbers presented in Table 1, while TPSA stands for Topological Polar Surface Area. In Table S5, the selected biomolecules under study have been labelled following the numbers presented in Table 1, while PAINS is an acronym for Pan Assay Interference Structures.

### 2.5. Conceptual DFT Studies

The calculated global reactivity descriptors estimated following the methodology presented in Materials and Methods Section (Conceptual DFT Studies subsection) together with the in-house developed CDFT software tool are displayed in Table 2 related to the molecular systems presented in Table 1. Although the HOMO energies are of the same order for all the molecules, the LUMO energies are smaller for the molecular systems 4, 5, 6, 16 and 17, thus implying different reactivity. Because global hardness is a direct measure of electron density deformation and chemical reactivity, which is related to the HOMO-LUMO gap, it can be seen that Pseudohypericin and Hypericin will be the most reactive molecules, while Actein and Rilpivirine will be the least reactive of all the molecules considered throughout this research. The electron donating power ω^−^ is more important than its electron accepting ω^+^ counterpart for all the ligands, which can explain their molecular and electronic structures. It is worth noting that the largest value for ω^−^ corresponds to Pseudohypericin and Hypericin. Moreover, when the values of ω^−^ and ω^+^ for each molecule are compared together with the net electrophilicity Δω±, it can be deduced that these last-mentioned molecules will have considerably different reactivity than the other systems.

## 3. Discussion

The selection of plant-based compounds based on their effectiveness on viral inhibition capacity was to omit other compounds and the study is reported [31,32]. This study found that the best docked binding poses for two quinone-type compounds adopted similar amino acid residues as inhibitor N3, i.e., hydrogen bonds with GLU166 and LEU141 residues, in total agreement with a recent report by Choudhary et al. [22]. In addition, they have well-accommodated binding sites, occupying the best binding pocket in a vertical position as the inhibitors. The non-covalent intermolecular interactions include electrostatic interactions, hydrogen bonds, hydrophobic, and van der Waals forces between the two molecules that affect the binding affinity towards the target protein.The lower the BE, the higher the stability of the complex.The efficiency of docking procedures is greatly improved by understanding the location of the binding site prior to docking actions. The hydrophobicity of the protein surface, conformational stability, chemical functional groups on the protein, and sizes of the protein are the parameters that influence the interaction between the protein and ligand.Furthermore, highly nucleophilic sites such as O-H groups in the ligands increase the interactions with M^pro^ protein, increasing the binding energy between ligands inside the 6LU7 receptor. During the interaction studies, it was noted that a hydrogen bond was noticed for amino acid GLU166 in both the compounds along with N3 inhibitor. Meanwhile, for LEU141, hydrogen bonding was noticed only in the anti-HIV compounds through other interactions in N3 inhibitor. The docking results suggest that the quinone compounds, Pseudohypericin and Hypericin, could be tested in vitro and in vivo as potent molecules against COVID-19. Several reports are available on the repurposing of anti-HIV compounds and drugs against M^pro^ of the novel SARS-CoV-2 virus by exploring an in silico computational evaluation, and reported many effective protease inhibitors as therapeutic agents for COVID-19 disease [31,32,33,34,35,36]. Nandet al. [31] initially carried out the sequence similarity analysis and screening using a deep learning approach, wherein two novel inhibitors were identified. Using a drug repurposing approach, Sang et al. [32] have determined that darunavir has the best binding affinity with SARS-CoV-1 3CL^pro^ and SARS-CoV-2. In another study, Barros et al. [33] grouped different ligand sets and confirmed that Saquinavir and Metaquine were effective against all receptors used in the in silico study.

In all cases, hydroxylation plays a vital role in interaction with COVID-19 main protease, as shown by the previously mentioned H-bond interaction type. Furthermore, reports suggested a positive role of 5-/7-hydroxyl derivatives flavonoid candidates by potential anti-H5N1 influenza A virus [37] and better inhibitory activity quercetin than morin in canine distemper virus inhibition [38]. Moreover, due to the presence of hydroxyl in gallate group, it has been demonstrated that EGCG ((-)-epigallocatechingallate) and ECG ((-)-epicatechingallate), compounds are the most effective free-radical scavengers compared to other standard antioxidants [39]. So, we can suggest that the inhibitory effect of quinone, flavonoids, phenolic, and alkaloids studied compounds against 6LU7 can be attributed to the presence of many –OH groups as the main ligand of the binding site. The literature shows that many anti-HIV protease inhibitor drugs, phyto-flavonoid compounds and small molecules, have been extensively used for in silico analysis against SARS-CoV-2 M^pro^ to date [40,41,42,43]. However, in the present study, anti-HIV bioactive medicinal compounds, which are plant-based, were subjected to in silico computational analysis wherein it was noted that Pseudohypericin was more potent than Hypericin as reported in Pitsillou et al. [41].

Hypericin and Pseudohypericin, Naphthodianthrones found in the extracts of *Hypericum perforatum* (St. John’s wort) are reported for their antibacterial, antidepressant, antipsoriatic, antiretroviral, antitumoral, antiviral, and photodynamic activities [44]. The pharmacokinetics of Hypericin and Pseudohypericin were previously analyzed and showed that both were low clearance drugs with a half-life of 41.7 h for Hypericin and 22.8 h for Pseudohypericin [45]. Furthermore, inhibitor N3 also showed a low bioavailability score of 0.17 and violated Lipinski’s rule. However, the anti-HIV drug, Rilpivirine showed no violations of Lipinski’s rule with a higher bioavailability score of 0.55. Recently, Hypericin from *H. perforatum* was reported as the most potent compound through computational investigation among Himalayan medicinal plant bioactives, which actively targets the inhibition of 3-chymotrypsin-like proteinase (3CL^pro^)/main proteases (M^pro^) and papain-like protease (PL^Pro^), which are involved in SARS-CoV-2 genome replication and transmission [46].

The electrophilicity ω index encompasses the equilibrium between the tendency of an electrophile to acquire extra electron density and a molecule’s resistance to exchanging electron density with the environment [47]. According to an electrophilicity ω scale for classifying organic molecules as strong, moderate, or marginal electrophiles (>1.5 eV for the first case, between 0.8 and 1.5 eV for the second case, and 0.8 eV for the last case) [48,49,50] and a review of Table S3, most of the most molecules may be regarded as strong electrophiles, with the exceptions of Robustaflavone, Procyanidin B2, (-)-Epicatechin-(4beta→8)-(-)-epigallocatechin, Actein, and Rilpivirine. These last molecular systems may be considered moderate electrophiles. A similar analysis may be conducted for the case of the nucleophilicity index N where according to a well-established scale presented earlier [48], the molecular systems one, two, and nine may be regarded as strong nucleophiles, while all the other molecules can be considered as moderate nucleophiles.

## 4. Materials and Methods

### 4.1. Ligand Preparation

In the present study, a total of 150 anti-HIV bioactive compounds from medicinal plants and 18 anti-HIV drugs were selected as the ligands, based on the review articles [25,26]. For the comparison, an inhibitor N3 was used as a docking comparison. All the compounds’ 3D structures (SDF files) were retrieved from the PubChem database (https://pubchem.ncbi.nlm.nih.gov/, accessed on 10 July 2020). The SDF files were then converted into PDB files by using online SMILES translator and structure file generator (https://cactus.nci.nih.gov/translate/, accessed on 10 July 2020).

### 4.2. Preparation of the Target Protein

The crystal structure of COVID-19 main protease (M^pro^) in complex with an inhibitor N3 (PDB: 6LU7) (2.16 Å) was used as a target protein to study the protein-ligand interaction. The 3D structure (PDB file) of protein was retrieved from the Research Collaboratory for Structural Bioinformatics Protein Data Bank (RCSB PDB) (https://www.rcsb.org/, accessed on 10 July 2020). The water molecules and ligands (inhibitor N3) were first removed from the protein structure using Discovery Studio Visualizer (Dassault Systems BIOVIA, 2016). The addition of hydrogen atoms and charges was carried out by the UCSF Chimera tool [27]. The computation of energy minimization and reconstruction of missing atoms wasconducted using Swiss-PDB Viewer. The processed protein was used for molecular docking studies.

### 4.3. Protein Structure Validation

The protein structure of M^pro^ of SARS-CoV-2 was further validated and evaluated for its chemical properties, bonds, and angles by the Ramachandran plot, which is generated by using PROCHECK via PDB sum database (http://www.ebi.ac.uk/thornton-srv/databases/pdbsum/Generate.html/, accessed on 11 June 2021). The obtained PROCHECK plot analysis represented that more than 90% of the amino acid residues are found within the most favoured regions of the protein (Figure S8).

### 4.4. Molecular Docking

Molecular docking was carried out to study the interaction of the ligand molecules with the target sites of M^pro^ protein using AutoDock Vina in PyRx software [28,29]. The whole target protein receptor was enclosed within the grid box dimension of 51.35 Å × 66.93 Å × 59.60 Å that coordinates with XYZ, respectively, at exhaustiveness of 100 poses. The confirmation of the least BE(expressed as kcal/mol) was considered the best docking pose. The protein-ligand interactions were visualized by using Discovery Studio Visualizer. The accuracy of the docking protocol was validated through re-docking (self-docking) of the compounds with the protein used during the study.

### 4.5. Molecular Dynamics (MD) Simulations

Based on the results obtained from molecular docking, the docked complex showing the best binding affinity and nonbonded interactions were further considered for molecular dynamics simulations. This was carried out using GROMACS v2021.2 (https://www.gromacs.org/, accessed on 1 January 2020). The force field applied for the simulation process was GROMOS96 43a1. The ligand topology files were generated using PRODRG software, to which the mol2 file of ligand was uploaded (http://davapc1.bioch.dundee.ac.uk/cgi-bin/prodrug/, accessed on 1 January 2020). SPC solvent model was used with a box shape of orthorhombic to determine the boundary conditions for solvation at a distance of 12 Å. The velocity-rescaling thermostat was employed in the MD simulations.For the MD run, the temperature was set at 300 K, pressure at1.0 bar, and simulation run time was set to 100 ns [30].

### 4.6. ADME (Absorption, Distribution, Metabolism and Excretion) Test

The selected potential molecules were subjected to the ADME test using the Swiss ADME tool (https://www.swissadme.ch/, accessed on 5 August 2020) to analyze lipophilicity, pharmacokinetics drug-likeness, and medicinal chemistry friendliness.

### 4.7. Conceptual DFT Studies

The Kohn-Sham (KS) approach [49] was used to determine the molecular energy, electronic density, and orbital energiesof a particular system, including the HighestOccupied Molecular Orbital (HOMO) and the Lowest Unoccupied Molecular Orbital (LUMO), using the CDFT or Conceptual Density Functional Theory variant of DFT [50]. The conformers of the compounds studied in this work were determined using Marvin View 17.15 from ChemAxon (http://www.chemaxon.com/, accessed on 1 January 2020) by using the entire MMFF94 force field to perform Molecular Mechanics calculations [51,52]. The Density FunctionalTightBinding (DFTBA)methodology [53] was considered for geometrypre optimization and frequency calculation.This step was necessary to guarantee that there were no imaginary frequencies within the energy surface, acommontest for the optimizedstructures’stability.Theestimationofthe chemical reactivity descriptors of the studied ligands was accomplished using the MN12SX/Def2TZVP/H_2_O model chemistry [54] on the optimized same level molecular structures because it has been shown that it fulfills the ‘Koopmans inDFT’ (KID)protocol [55].Gaussian16 [53] and the SMD solvent model [56] were considered for the determinations.This model chemistry is based on applying the MN12SX density functional in connection to the Def2TZVP basis set. The charge of the molecules is equal to zero and considering the corresponding negative and positive ions in the doublet spin state.

The definitions for the global reactivity descriptors are [57]: Electronegativity as $\chi \approx \frac{1}{2}\;\left(\varepsilon_{H}+\varepsilon_{L}\right)$, Global Hardness as $\eta \approx \left(\varepsilon_{L}-\varepsilon_{H}\right)$, Electrophilicity as $\omega \approx \frac{{\left(\varepsilon_{H}+\varepsilon_{L}\right)}^{2}}{4\;\left(\varepsilon_{L}-\varepsilon_{H}\right)}$, Electrodonating Power as $\omega^{-}\approx \frac{{\left(3\varepsilon_{H}+\varepsilon_{L}\right)}^{2}}{16\;\eta }$, Electroaccepting Power as $\omega^{+}\approx \frac{{\left(\varepsilon_{H}+3\varepsilon_{L}\right)}^{2}}{16\;\eta }$, and Net Electrophilicity as $\Delta \omega^{\pm }=\omega^{+}-\left(-\omega^{-}\right)=\omega^{+}+\omega^{-}$, being $\varepsilon_{H}$ and $\varepsilon_{L}$, the energies of the HOMO and LUMO orbitals, respectively. These global reactivity descriptors that arise from Conceptual DFT have been complemented by a Nucleophilicity Index N [48] that considers the value of the HOMO energy obtained by means of the KS scheme using an arbitrary shift of the origin with tetracyanoethylene (TCE) as a reference.

## 5. Conclusions

The study focused on the virtual screening of 150 anti-HIV bioactive compounds from medicinal plants through molecular docking against SARS-CoV-2 M^pro^ to predict the best possible compound that may be utilized for in vitro and in vivo studies as a potential candidate against COVID-19 disease. The study results showed that among the compounds screened, 16 compounds exhibited higher binding energies than a reference molecule and the standard drugs analyzed in the study. The lowest binding affinity of −10.2 kcal/mol was observed in Pseudohypericin with four amino acid residual interactions (LEU141, GLU166, ARG188 and GLN192), followed by Hypericin (−10.1 kcal/mol). The ADME analysis of Pseudohypericin and Hypericin recorded a low BA score of 0.17, violating Lipinski’s rule. Pseudohypericin showed a lower binding energy value than Hypericin, and the MD simulations estimated that the Pseudohypericin-protein complex was stable throughout the simulation. We can conclude that the quinone compound, Pseudohypericin, possesses the potential to be tested in vitro and in vivo prior to conducting clinical trials as potent biomolecules against COVID-19 disease.

## Figures and Tables

**Table 1:** Molecular docking results of the anti-HIV bioactive compounds against M^pro^ of SARS-CoV-2.

Sl. No.	Name of Compound	Name of Plants	Class	Binding Energy (kcal/mol)	Interaction of Residues with Hydrogen Bond
1	Pseudohypericin	*Hypericum perforatum*	Quinone	−10.2	LEU141, GLU166, ARG188, GLN192
2	Hypericin	*Hypericum perforatum*	Quinone	−10.1	HIS41, LEU141, ASN142, CYS145, GLU166
3	Robustaflavone	*Rhus succedanea*	Flavonoid	−9.7	HIS163, HIS164
4	Procyanidin B2	*Maytenus senegalensis*	Flavonoid	−9.3	THR26, HIS41, LEU141, GLU166
5	(-)-Epicatechin-(4beta→8)-(-)-epigallocatechin	*Maytenus senegalensis*	Phenolic	−9.3	LEU141, SER144, GLU166
6	(-)-Epicatechin(4.beta.→8)(-)-4′-methylepigallocatechin	*Maytenus senegalensis*	Phenolic	−9.2	THR26, SER144, GLU166
7	Agathisflavone	*Rhus succedanea*	Flavonoid	−9.2	GLU166
8	Hinokiflavone	*Rhus succedanea*	Flavonoid	−9.2	GLY109, GLN110
9	Michellamine B	*Ancistrocladuskorupensis*	Alkaloid	−9.2	LEU220, ARG222
10	Rhusflavanone	*Rhus succedanea*	Flavonoid	−9.1	GLN189
11	GB-1a 7′-glucoside	*Garcinia multiflora*	Flavonoid	−9.1	THR26, GLU166
12	Quercetin 3-O-(2″galloyl)-alpha-L-arabinopyranoside	*Acer okamotoanum*	Flavonoid	−9.0	ASN142, GLU166
13	Wikstrol B	*Wikstroemia indica*	Flavonoid	−9.0	HIS41, HIS163
14	Morelloflavone	*Garcinia multiflora*	Flavonoid	−8.9	PHE140, ASN142, GLN189, THR190
15	Quercitrin	*Acer okamotoanum*	Flavonoid	−8.9	LEU141, ASN142, HIS163, GLU166
16	Actein	*Cimicifuga racemosa*	Terpene	−8.9	ARG131, GLY195
17	Rilpivirine		Drug	−8.9	SER46

**Table 2:** CDFT Global Reactivity Descriptors of the Selected Potential Biomolecules.

Sl. No.	Homo	Lumo	Homo-Lumo Gap	*χ*	*η*	*ω*	S	N	*ω* ^−^	*ω* ^+^	Δ*ω*±
1	−5.29	−3.33	1.96	4.31	1.96	4.74	0.51	3.51	11.76	7.45	19.20
2	−5.27	−3.28	1.99	4.27	1.99	4.59	0.50	3.52	11.44	7.17	18.61
3	−6.16	−2.43	3.73	4.30	3.73	2.47	0.27	2.63	7.33	3.03	10.36
4	−5.90	−0.68	5.21	3.29	5.21	1.04	0.19	2.90	4.05	0.76	4.80
5	−5.94	−0.68	5.26	3.31	5.26	1.04	0.19	2.85	4.07	0.76	4.83
6	−5.94	−0.68	5.26	3.31	5.26	1.04	0.19	2.85	4.07	0.76	4.83
7	−6.12	−2.42	3.70	4.27	3.70	2.46	0.27	2.67	7.29	3.02	10.32
8	−6.24	−2.46	3.78	4.35	3.78	2.50	0.26	2.55	7.42	3.07	10.49
9	−5.43	−1.70	3.73	3.56	3.73	1.80	0.27	3.37	5.42	1.85	7.27
10	−6.11	−1.87	4.24	3.99	4.24	1.87	0.24	2.69	6.01	2.03	8.04
11	−6.25	−1.98	4.27	4.11	4.27	1.98	0.23	2.55	6.29	2.18	8.47
12	−6.14	−2.52	3.62	4.33	3.62	2.59	0.28	2.65	7.56	3.23	10.79
13	−5.91	−2.41	3.50	4.16	3.50	2.47	0.29	2.89	7.23	3.08	10.31
14	−6.06	−2.28	3.78	4.17	3.78	2.31	0.26	2.73	6.93	2.76	9.70
15	−6.10	−2.38	3.72	4.24	3.72	2.42	0.27	2.69	7.19	2.95	10.14
16	−6.60	−0.33	6.27	3.46	6.27	0.96	0.16	2.19	4.04	0.57	4.51
17	−6.49	−0.22	6.27	3.35	6.28	0.90	0.16	2.30	3.86	0.51	4.37

Note: χ—Electronegativity; η—Global Hardness; ω—Electrophilicity; S—Global Softness; N—Nucleophilicity; ω^−^—Electrodonating Power; ω^+^—Electroaccepting Power; Δ*ω*±—Net Electrophilicity. All the descriptors are expressed in eV, with the exception of S, which is expressed in eV^−1^.

## Data Availability

The data presented in this study are available within the manuscript.

## Supplementary Materials

The following supporting information can be downloaded at: https://www.mdpi.com/article/10.3390/molecules27238288/s1, Figure S1. Anti-HIV compounds Robustaflavone and Procyanidin B2 docked with M^pro^ of SARS-CoV-2. Figure S2. Anti-HIV compounds (-)-Epicatechin-(4beta→8)-(-)-epigallocatechin and (-)-Epicatechin(4.beta.→8)(-)-4′-methylepigallocatechin docked with M^pro^ of SARS-CoV-2. Figure S3. Anti-HIV compounds Agathisflavone and Hinokiflavone docked with M^pro^ of SARS-CoV-2. Figure S4. Anti-HIV compounds Michellamine B and Rhusflavanone docked with M^pro^ of SARS-CoV-2. Figure S5. Anti-HIV compounds GB-1a 7′-glucoside and Quercetin 3-O-(2″galloyl)-alpha-L-arabinopyranoside docked with M^pro^ of SARS-CoV-2. Figure S6. Anti-HIV compounds Wikstrol B and Morelloflavone docked with M^pro^ of SARS-CoV-2. Figure S7. Anti-HIV compounds Quercitrin and Actein docked with M^pro^ of SARS-CoV-2. Figure S8. Ramachandran plot for the model of M^pro^ protein structure of SARS-CoV-2 generated by PROCHECK. Red color region denotes residues of the protein in the most favored regions, brown color denotes residues in the additional allowed regions, and yellow indicates residues in the generously allowed regions. Table S1. Docking results of selected anti-HIV bioactive compounds against M^pro^ of SARS-CoV-2 (PDB: 6LU7). Table S2. Docking results of selected drugs and inhibitor against M^pro^ of SARS-CoV-2 (PDB: 6LU7). Table S3. Lipophilicity and Pharmacokinetics of the Selected Potential Biomolecules. Table S4. Drug likeness of the Selected Potential Biomolecules. Table S5. Medicinal Chemistry Friendliness of the Selected Potential Biomolecules.
