# Supplementary material for: Exploration of Anti-HIV Phytocompounds against SARS-CoV-2 Main Protease: Structure-Based Screening, Molecular Simulation, ADME Analysis and Conceptual DFT Studies
Source: Molecules. 2022 Nov 28;27(23):8288. doi: 10.3390/molecules27238288 (PMC9736867; doi:10.3390/molecules27238288)
Supplement: Supplementary file 1 [file molecules-27-08288-s001.zip › molecules-1937353-supplementary.pdf]

Supplementary Materials

Exploration of Anti-HIV Phytocompounds against SARS-CoV-2 Main Protease: Structure-based Screening, Molecular Simulation and ADMET Analysis

Mahadevamurthy Murali[1], Hittanahallikoppal Gajendramurthy Gowtham[2], Natarajamurthy Shilpa[3], Hemanth Kumar Naguvanahalli Krishnappa[1], Ana E. Ledesma[4], Anisha S. Jain[5], Ali A. Shati[6], Mohammad Y. Alfaifi[6], Serag Eldin I. Elbehairi[6,7], Raghu Ram Achar[8], Ekaterina Silina[9], Victor Stupin[9], Joaquín Ortega-Castro[10], Juan Frau[10], Norma Flores-Holguín[11], Kestur Nagaraj Amruthesh[1,*], Chandan Shivamallu[12,*], Shiva Prasad Kollur[13,*] and Daniel Glossman-Mitnik [11]

[1] Department of Studies in Botany, University of Mysore, Manasagangotri, Mysore 570006, Karnataka, India
[2] Department of PG Studies in Biotechnology, Nrupathunga University, Nrupathunga Road, Bangalore 560001, India
[3] Department of Studies in Microbiology, University of Mysore, Manasagangotri, Mysore 570006, Karnataka, India
[4] Centro de Investigaciónen Biofísica Aplicada y Alimentos, Universidad Nacional de Santiago del Estero (CIBAAL-UNSE-CONICET), FCEyN, Santiago del Estero 4206, Argentina
[5] Department of Microbiology, JSS Academy of Higher Education and Research, Mysuru 570015, Karnataka, India
[6] King Khalid University, Faculty of Science, Biology Department, Abha 9004, Saudi Arabia
[7] Cell Culture Lab, Egyptian Organization for Biological Products and Vaccines (VACSERA Holding Company), 51 Wezaret El-Zeraa St., Agouza, Giza, Egypt
[8] Division of Biochemistry, School of Life Sciences, JSS Academy of Higher Education and Research, Mysuru – 570 015, Karnataka, India
[9] Department of Hospital Surgery, N.I. Pirogov Russian National Research Medical University, Mascow, Russia
[10] Departament de Química, Facultat de Ciencies, Universitat de les Illes Balears, Palma de Malllorca, E-07122, Spain
[11] Laboratorio Virtual NANOCOSMOS, Departamento de Medio Ambiente y Energía, Centro de Investigaciónen Materiales Avanzados, Chihuahua, Chih 31136, México
[12] Department of Biotechnology and Bioinformatics, JSS Academy of Higher Education and Research, Mysuru-570015, Karnataka, India
[13] School of Physical Sciences, Amrita Vishwa Vidyapeetham, Mysuru Campus, Mysuru - 570026, Karnataka, India
*Correspondence: shivachemist@gmail.com (SPK), chandans@jssuni.edu.in (CS), dr.knamruthesh@botany.uni-mysore.ac.in (KNA)

This document includes:

Figures S1 to S8
Tables S1 to S5

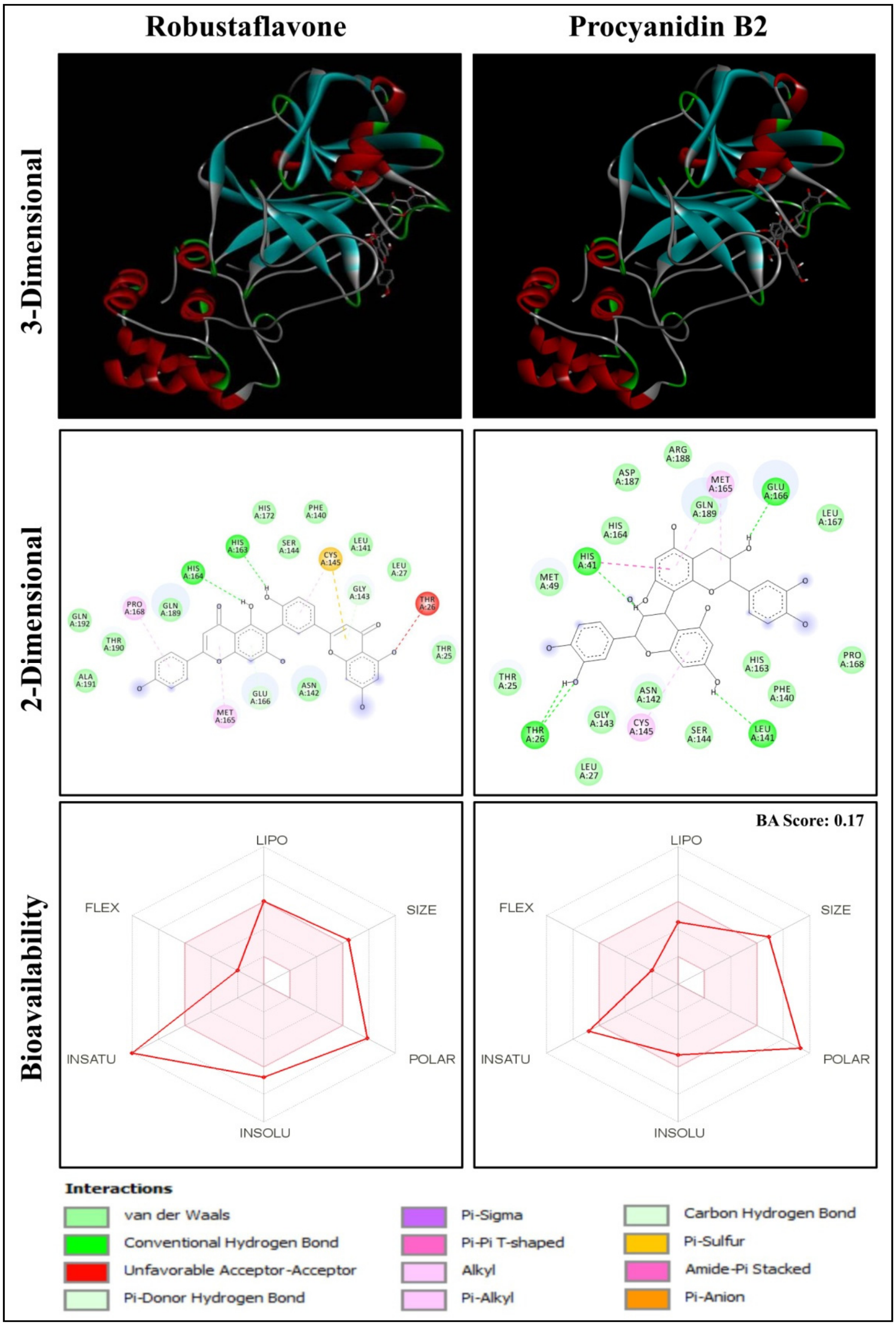

Figure S1. Anti-HIV compounds Robustaflavone and Procyanidin B2 docked with M^pro of SARS-CoV-2

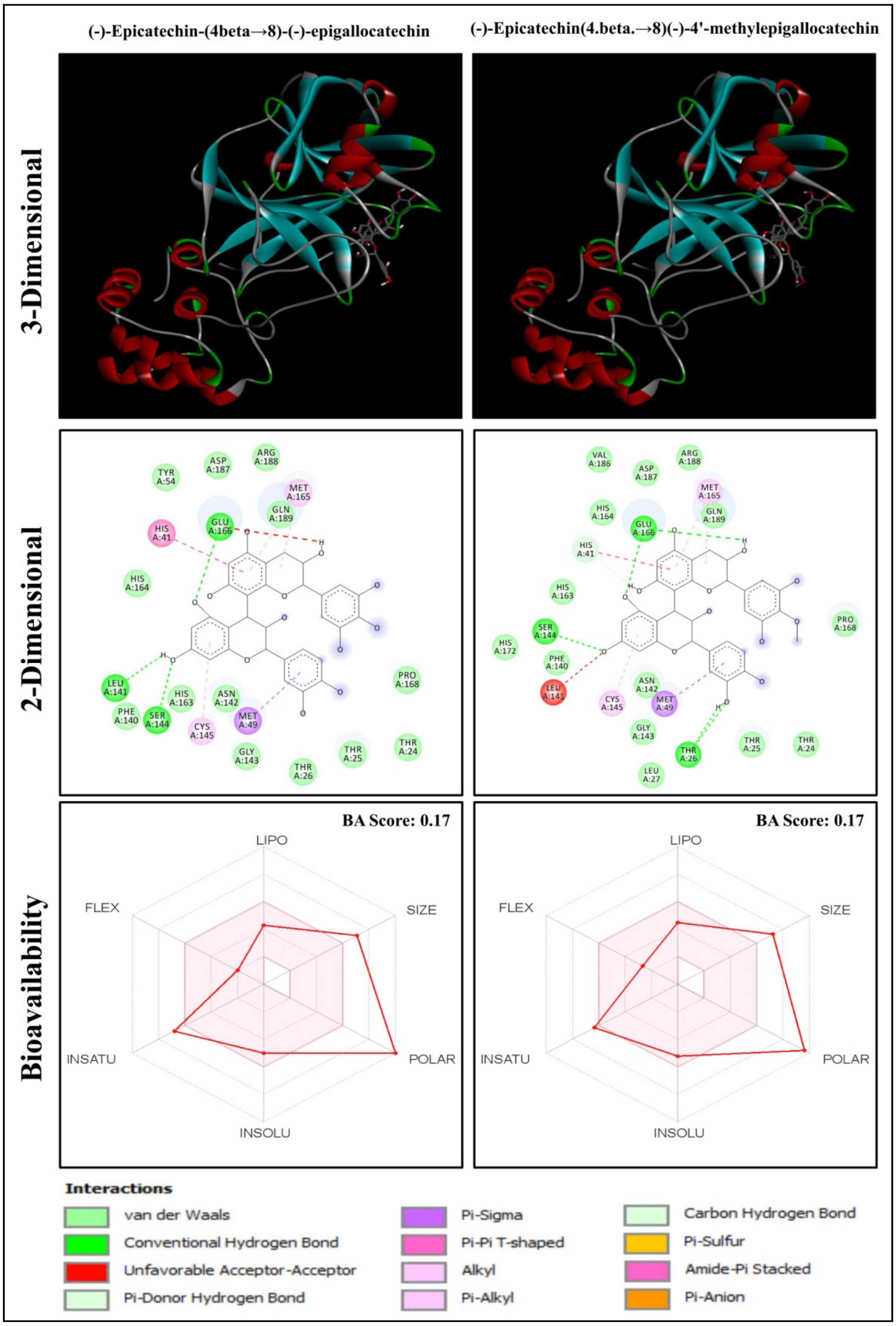

Figure S2. Anti-HIV compounds (-)-Epicatechin-(4beta→8)-(-)-epigallocatechin and (-)-Epicatechin(4.beta.→8)(-)-4'-methylepigallocatechin docked with M^{pro} of SARS-CoV-2

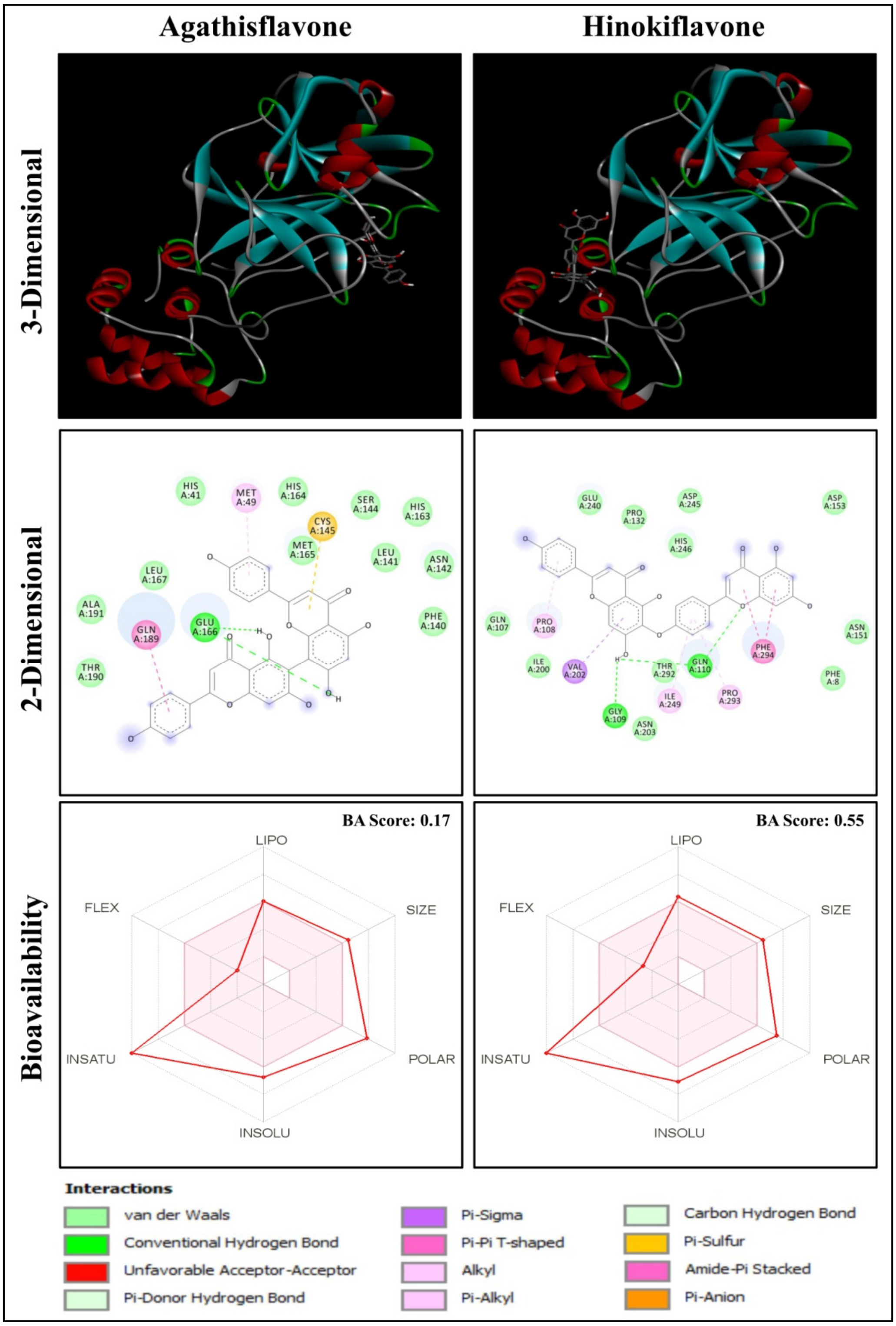

Figure S3. Anti-HIV compounds Agathisflavone and Hinokiflavone docked with M^{Pro} of SARS-CoV-2

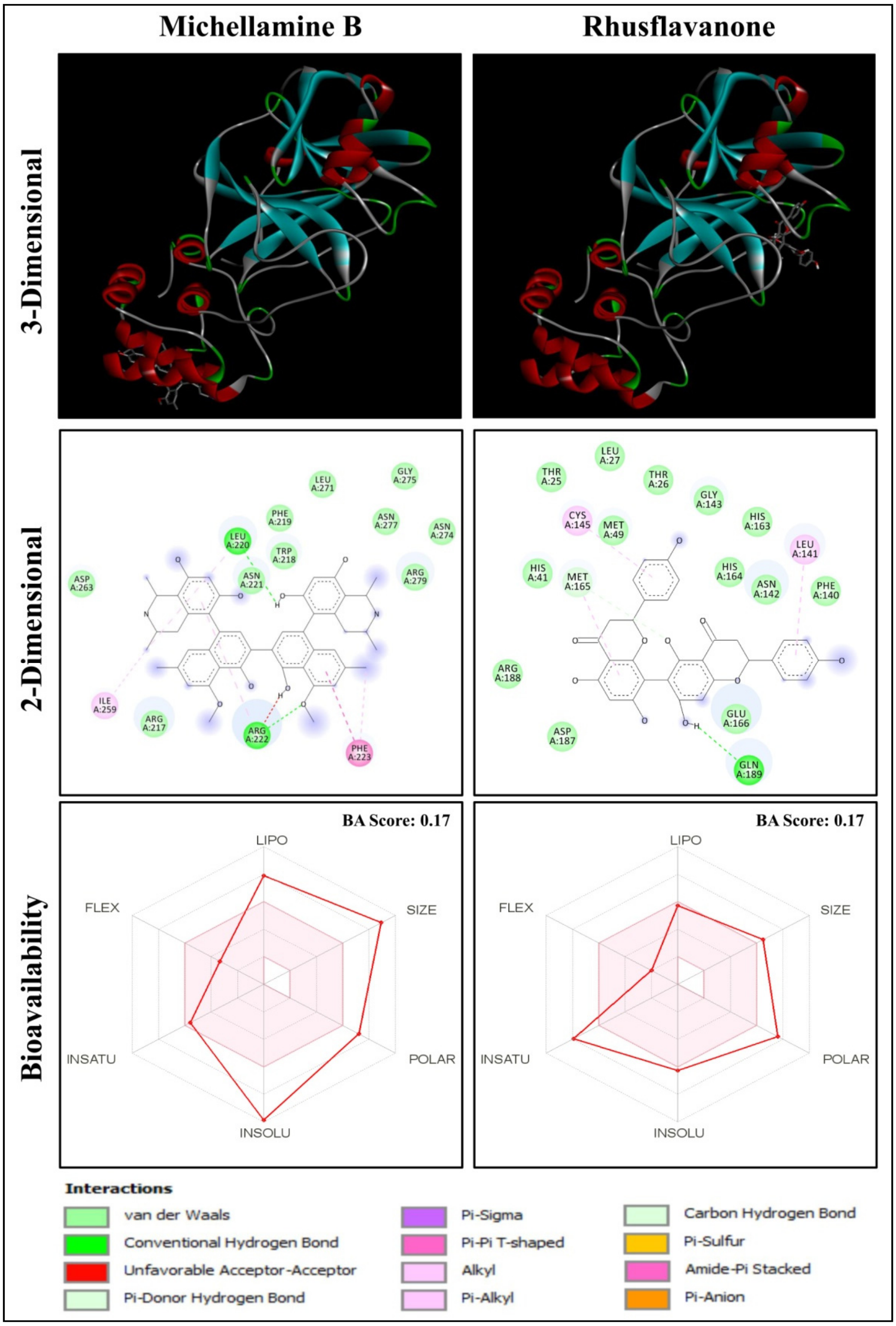

Figure S4. Anti-HIV compounds Michellamine B and Rhusflavanone docked with Mpro of SARS-CoV-2

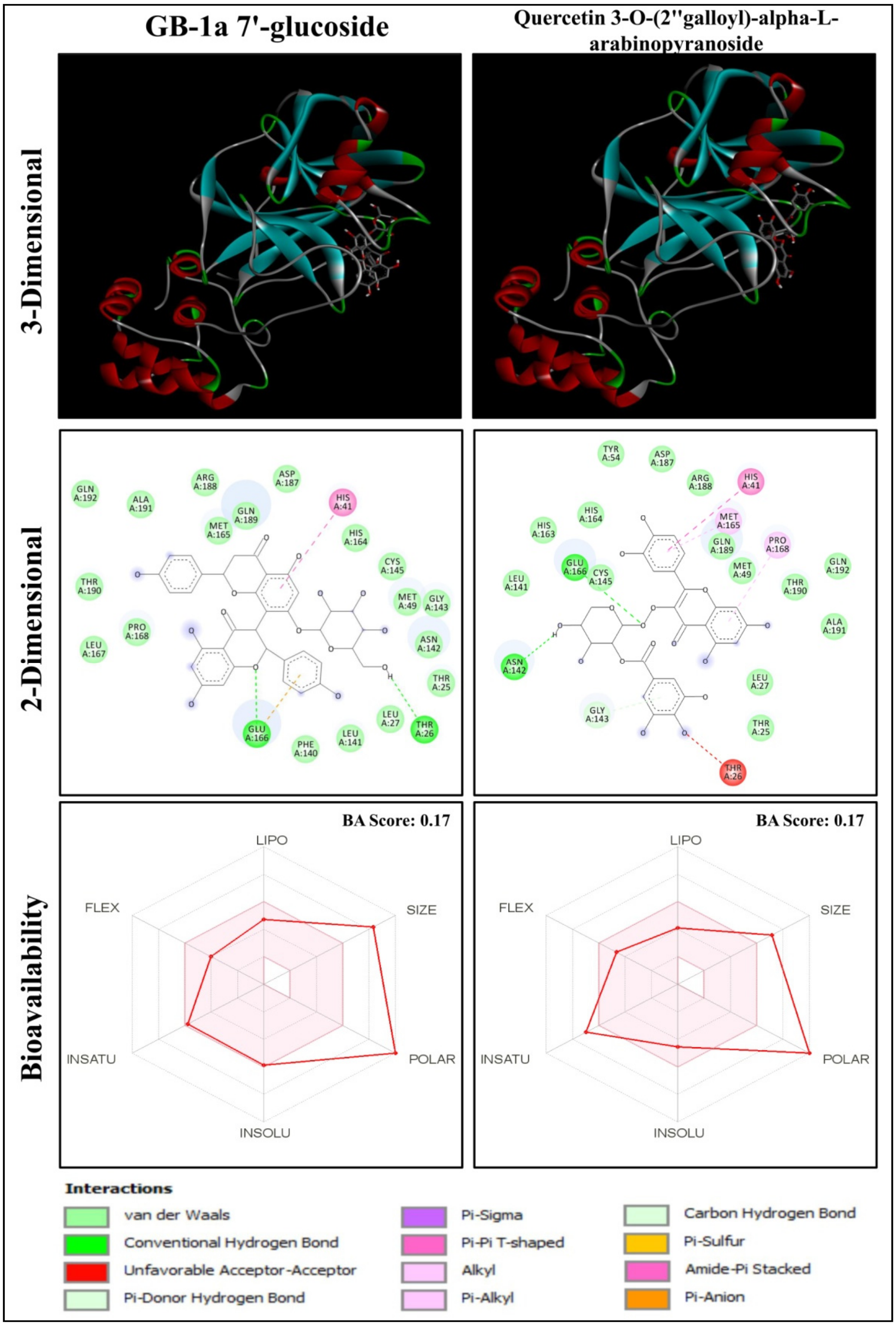

Figure S5. Anti-HIV compounds GB-1a 7'-glucoside and Quercetin 3-O-(2''galloyl)-alpha-L-arabinopyranoside docked with M^pro^ of SARS-CoV-2

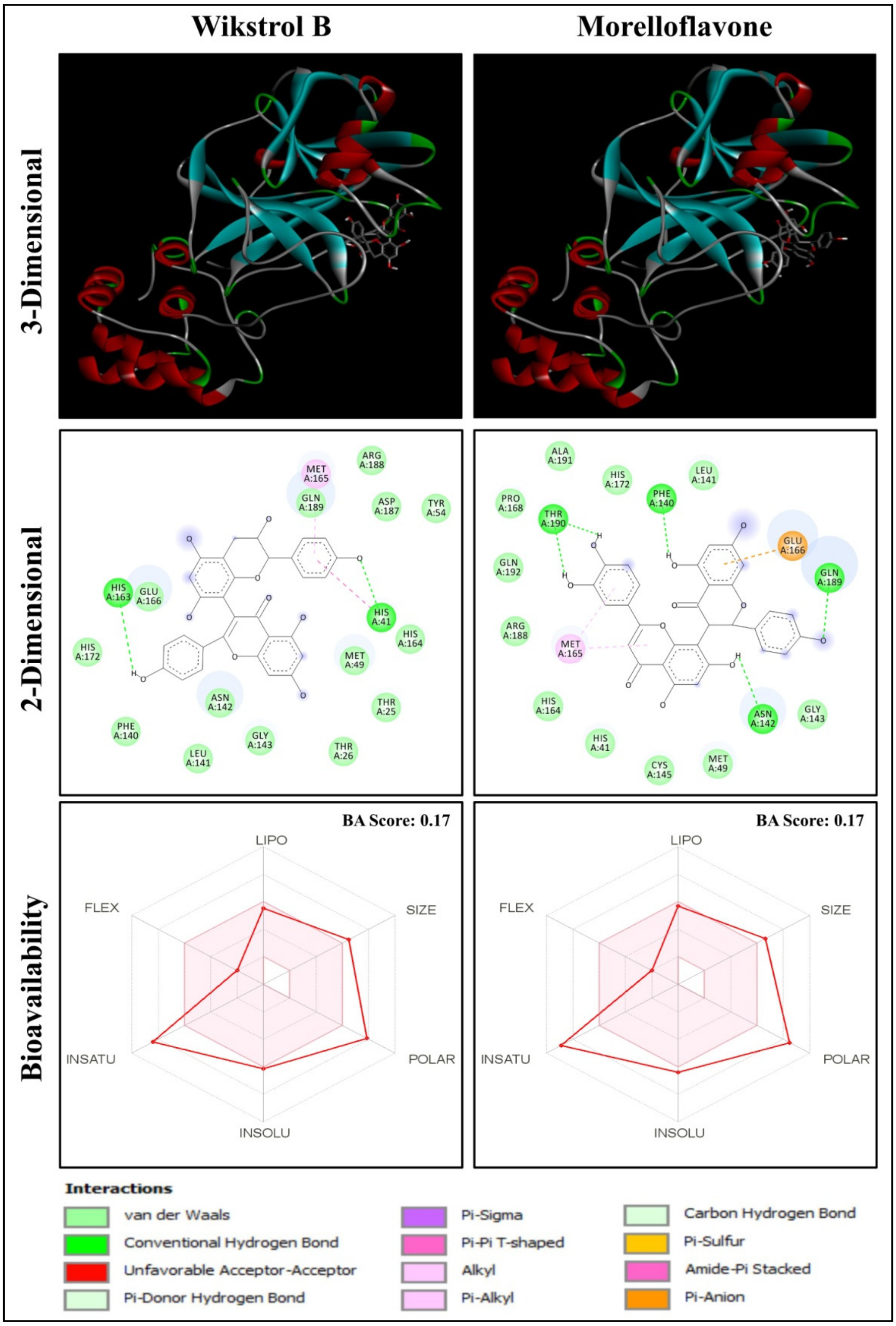

Figure S6. Anti-HIV compounds Wikstrol B and Morelloflavone docked with M^{pro} of SARS-CoV-2

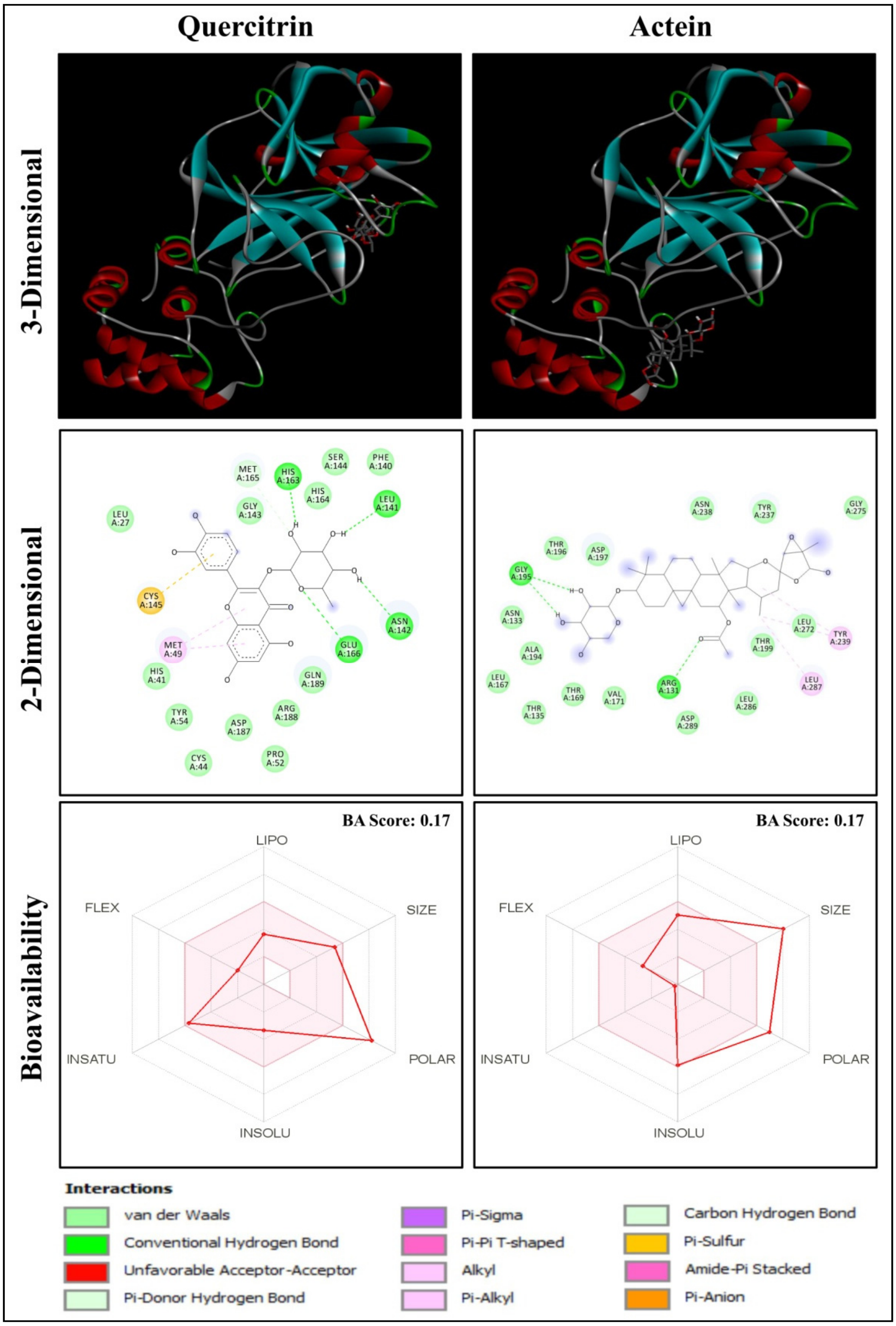

Figure S7. Anti-HIV compounds Quercitrin and Actein docked with M^{pro} of SARS-CoV-2

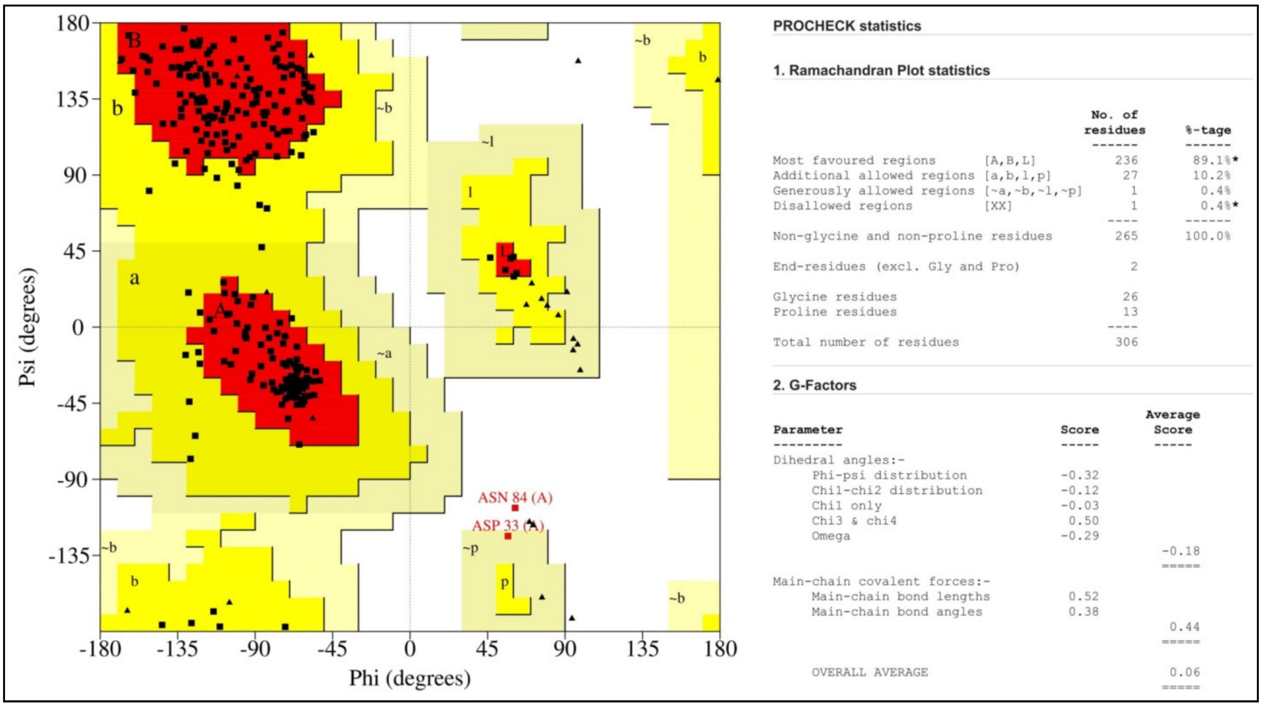

Figure S8. Ramachandran plot for the model of M^pro^ protein structure of SARS-CoV-2 generated by PROCHECK. Red color region denotes residues of the protein in the most favored regions, brown color denotes residues in the additional allowed regions, and yellow indicates residues in the generously allowed regions

Table S1. Docking results of selected anti-HIV bioactive compounds against M^pro of SARS-CoV-2 (PDB: 6LU7)

Sl. No.	Name of Compound	Canonical SMILES	Name of Plants	Binding energy (kcal/mol)	Group
1.	Agathisflavone	C1=CC(=CC=C1C2=CC(=O)C3=C(O2)C=C(C(=C3O)C4=C(C=C(C5=C4OC(=CC5=O)C6=CC=C(C=C6)O)O)O)O)O	*Rhus succedanea*	-9.2	Flavonoid
2.	Robustaflavone	C1=CC(=CC=C1C2=CC(=O)C3=C(O2)C=C(C(=C3O)C4=C(C=CC(=C4)C5=CC(=O)C6=C(C=C(C=C6O5)O)O)O)O)O	*Rhus succedanea*	-9.7	Flavonoid
3.	Hinokiflavone	C1=CC(=CC=C1C2=CC(=O)C3=C(O2)C=C(C(=C3O)OC4=CC=C(C=C4)C5=CC(=O)C6=C(C=C(C=C6O5)O)O)O)O	*Rhus succedanea*	-9.2	Flavonoid
4.	Rhusflavanone	C1C(OC2=C(C1=O)C(=C(C(=C2)O)C3=C4C(=C(C=C3O)O)C(=O)CC(O4)C5=CC=C(C=C5)O)O)C6=CC=C(C=C6)O	*Rhus succedanea*	-9.1	Flavonoid
5.	Succedaneaflavanone	C1C(OC2=C(C1=O)C(=C(C(=C2)O)C3=C(C4=C(C=C3O)OC(CC4=O)C5=CC=C(C=C5)O)O)O)C6=CC=C(C=C6)O	*Rhus succedanea*	-8.4	Flavonoid
6.	Volkensiflavone	C1=CC(=CC=C1C2C(C(=O)C3=C(C=C(C=C3O2)O)O)C4=C(C=C(C5=C4OC(=CC5=O)C6=CC=C(C=C6)O)O)O)O	*Garcinia multiflora*	-8.3	Flavonoid
7.	Morelloflavone	C1=CC(=CC=C1C2C(C(=O)C3=C(C=C(C=C3O2)O)O)C4=C(C=C(C5=C4OC(=CC5=O)C6=CC(=C(C=C6)O)O)O)O)O	*Garcinia multiflora*	-8.9	Flavonoid
8.	GB-1a	C1C(OC2=C(C(=CC(=C2C1=O)O)O)C3C(OC4=CC(=CC(=C4C3=O)O)O)C5=CC=C(C=C5)O)C6=CC=C(C=C6)O	*Garcinia multiflora*	-8.2	Flavonoid
9.	GB-1a 7'-glucoside	C1C(OC2=C(C1=O)C(=CC(=C2C3C(OC4=CC(=CC(=C4C3=O)O)O)C5=CC=C(C=C5)O)OC6C(C(C(C(O6)CO)O)O)O)O)C7=CC=C(C=C7)O	*Garcinia multiflora*	-9.1	Flavonoid
10.	GB-2a	C1C(OC2=C(C(=CC(=C2C1=O)O)O)C3C(OC4=CC(=CC(=C4C3=O)O)O)C5=CC=C(C=C5)O)C6=CC(=C(C=C6)O)O	*Garcinia multiflora*	-8.4	Flavonoid
11.	Michellamine B	CC1CC2=C(C(=CC(=C2C(N1)C)O)O)C3=CC(=C(C4=C3C=C(C=C4OC)C)O)C5=C(C6=C(C=C(C=C6OC)C)C(=C5)C7=C8CC(NC(C8=C(C=C7O)O)C)C)O	*Ancistrocladuskorupensis*	-9.2	Alkaloid
12.	Papaverine	COC1=C(C=C(C=C1)CC2=NC=CC3=CC(=C(C=C32)OC)OC)OC	*Papaver somniferum*	-6.9	Alkaloid
13.	Imperatorin	CC(=CCOC1=C2C(=CC3=C1OC=C3)C=CC(=O)O2)C	*Aegle marmelos*	-7.1	Coumarin
14.	Xanthotoxol	C1=CC(=O)OC2=C(C3=C(C=CO3)C=C21)O	*Aegle marmelos*	-6.4	Coumarin
15.	Xanthotoxin	COC1=C2C(=CC3=C1OC=C3)C=CC(=O)O2	*Aegle marmelos*	-6.4	Coumarin
16.	Auraptene	CC(=CCCC(=CCOC1=CC2=C(C=C1)C=CC(=O)O2)C)C	*Aegle marmelos*	-6.8	Coumarin

17.	Protopine	CN1CCC2=CC3=C(C=C2C(=O)CC4=C(C1)C5=C(C=C4)OCO5)OCO3	*Argemone mexicana*	-7.9	Alkaloid
18.	Berberine	COC1=C(C2=C[N+]3=C(C=C2C=C1)C4=CC5=C(C=C4CC3)OCO5)OC	*Argemone mexicana*	-7.3	Alkaloid
19.	Daidzein	C1=CC(=CC=C1C2=COC3=C(C2=O)C=CC(=C3)O)O	*Butea monosperma*	-7.0	Flavonoid
20.	Xanthopurpurin	C1=CC=C2C(=C1)C(=O)C3=C(C2=O)C(=CC(=C3)O)O	*Rubia cordifolia*	-7.1	Quinone
21.	Betulinic acid	CC(=C)C1CCC2(C1C3CCC4C5(CCC(C(C5CCC4(C3(CC2)C)C)(C)C)O)C)C(=O)O	*Syzygiumclaviflorum, Vatica cinerea*	-7.5	Terpene
22.	Oleanolic acid	CC1(CCC2(CCC3(C(=CCC4C3(CCC5C4(CCC(C5(C)C)O)C)C)C2C1)C)C(=O)O)C	*Rosa woodsii, Prosopis glandulosa, Phoradendronjuniperinum, Syzygiumclaviflorum, Hyptiscapitata, Ternstroemiagymnanthera*	-8.4	Terpene
23.	Pomolic acid	CC1CCC2(CCC3(C(=CCC4C3(CCC5C4(CCC(C5(C)C)O)C)C)C2C1(C)O)C)C(=O)O	*Prosopis glandulosa, Phoradendron juniperinum, Syzygiumclaviflorum, Hyptis capitata*	-7.6	Terpene
24.	Ursolic acid	CC1CCC2(CCC3(C(=CCC4C3(CCC5C4(CCC(C5(C)C)O)C)C)C2C1C)C)C(=O)O	*Prosopis glandulosa, Phoradendron juniperinum, Syzygiumclaviflorum, Hyptiscapitata, Crataeguspinnatifida, Vaticacinerea*	-7.9	Terpene
25.	Xanthohumol	CC(=CCC1=C(C(=C(C=C1O)OC)C(=O)C=CC2=CC=C(C=C2)O)O)C	*Humulus lupulus*	-7.4	Flavonoid
26.	Cordatolide A	CC1C(OC2=C(C1O)C3=C(C(=CC(=O)O3)C)C4=C2C=CC(O4)(C)C)C	*Calophyllum* species	-7.6	Coumarin
27.	Cordatolide B	CC1C(OC2=C(C1O)C3=C(C(=CC(=O)O3)C)C4=C2C=CC(O4)(C)C)C	*Calophyllum* species	-7.8	Coumarin
28.	Calanolide A	CCCC1=CC(=O)OC2=C1C3=C(C=CC(O3)(C)C)C4=C2C(C(C(O4)C)C)O	*Calophyllumlanigerum*	-7.8	Coumarin
29.	Oblongulide	CC=C(C)C(=O)C1=C(C2=C(C3=C1OC(=O)C=C3C)OC(C=C2)(C)C)OC	*Calophyllum* species	-6.8	Coumarin

30.	Thwaitesixanthone	CC1(C=CC2=C(O1)C=CC3=C2C(=O)C4=C(C5=C(C=C4O3)OC(C=C5)(C)C)O)C	*Calophyllum* species	-8.1	Coumarin
31.	Calothwaitesixanthone	CC(=CCC1=C(C2=C(C=C1O)OC3=C(C2=O)C4=C(C=C3)OC(C=C4)(C)C)O)C	*Calophyllum* species	-8.2	Coumarin
32.	Calozeyloxanthone	CC1=CC2C(CC1)C(OC3=C2C4=C(C=C3)OC5=CC(=CC(=C5C4=O)O)O)(C)C	*Calophyllum* species	-8.2	Coumarin
33.	Cordato-oblongic acid	CC1C(OC2=C(C1=O)C(=C3C=CC(OC3=C2C(C)CC(=O)O)(C)C)O)C	*Calophyllum* species	-7.7	Coumarin
34.	Cordato-oblongic acid methyl ester	CC1C(OC2=C(C1=O)C(=C3C=CC(OC3=C2C(C)CC(=O)OC)(C)C)O)C	*Calophyllum* species	-7.2	Coumarin
35.	Isocordato-oblongic acid methyl ester	CC1C(OC2=C(C1=O)C(=C3C=CC(OC3=C2C(C)CC(=O)OC)(C)C)O)C	*Calophyllum* species	-6.9	Coumarin
36.	Inophyllum A	CC1C(OC2=C(C1O)C3=C(C(=CC(=O)O3)C4=CC=CC=C4)C5=C2C=CC(O5)(C)C)C	*Calophyllum* species	-8.3	Coumarin
37.	Bergenin	COC1=C(C=C2C(=C1O)C3C(C(C(C(O3)CO)O)O)OC2=O)O	*Peltophorumafricanum*	-6.9	Glycoside
38.	Catechin	C1C(C(OC2=CC(=CC(=C21)O)O)C3=CC(=C(C=C3)O)O)O	*Peltophorumafricanum, Detariummicrocarpum*	-7.1	Flavonoid
39.	Garciosaterpene A	CC(CCC=C(C)C(=O)O)C1CCC2(C1=CCC3C2(CCC4C3(CCC(C4(C)C)OC(=O)C)C)C)C	*Garcinia speciosa*	-7.4	Terpene
40.	Garciosaterpene B	CC(CCC=C(C)C(=O)O)C1CCC2(C1=CCC3C2(CCC4C3(CCC(C4(C)C)O)C)C)C	*Garcinia speciosa*	-7.7	Terpene
41.	Garciosaterpene C	CC(CCC=C(C)C(=O)O)C1CCC2(C1=CCC3C2(CCC4C3(CCC(=O)C4(C)C)C)C)C	*Garcinia speciosa*	-7.6	Terpene
42.	Garciosaphenone A	CC(=CCCC(=CCC1=C(C(=C(C=C1O)O)CC=C(C)CCC=C(C)C)C(=O)C2=C(C=C(C=C2O)O)O)C)C	*Garcinia speciosa*	-6.7	Terpene
43.	Hypericin	CC1=CC(=O)C2=C(C3=C(C=C(C4=C3C5=C2C1=C6C(=CC(=O)C7=C(C8=C(C=C(C4=C8C5=C67)O)O)O)C)O)O)O	*Hypericum perforatum*	-10.1	Quinone
44.	Pseudohypericin	CC1=CC(=O)C2=C(C3=C(C=C(C4=C3C5=C2C1=C6C(=CC(=O)C7=C(C8=C(C=C(C4=C8C5=C67)O)O)O)CO)O)O)O	*Hypericum perforatum*	-10.2	Quinone
45.	Linearol	CC(=O)OCC1(C(CCC2(C1CC(C34C2CCC(C3)C(=C)C4)O)C)O)C	*Sideritis* spp.	-7.1	Terpene
46.	Rosmarinic acid	C1=CC(=C(C=C1CC(C(=O)O)OC(=O)C=CC2=CC(=C(C=C2)O)O)O)O	*Melissa officinalis*	-7.7	Phenolic
47.	Swertifrancheside	COC1=CC(=C2C(=C1)OC3=C(C=C(C(=C3C2=O)O)C4=C5C(=C(C(=C4O)C6C(C(C(C(O6)CO)O)O)O)O)C(=O)C=C(O5)C7=CC(=C(C=C7)O)O)O)O	*Swertia franchetiana*	-8.4	Xanthone

48.	(-)-Epicatechin	C1C(C(OC2=CC(=CC(=C21)O)O)C3=CC(=C(C=C3)O)O)O	*Detariummicrocarpum, Camellia sinensis*	-7.0	Flavonoid
49.	(-)-Epicatechin gallate	C1C(C(OC2=CC(=CC(=C21)O)O)C3=CC(=C(C=C3)O)O)OC(=O)C4=CC(=C(C(=C4)O)O)O	*Detariummicrocarpum, Camellia sinensis*	-8.3	Flavonoid
50.	(+)-Gallocatechin	C1C(C(OC2=CC(=CC(=C21)O)O)C3=CC(=C(C(=C3)O)O)O)O	*Croton draconoides*	-7.2	Flavonoid
51.	(-)-Epigallocatechin	C1C(C(OC2=CC(=CC(=C21)O)O)C3=CC(=C(C(=C3)O)O)O)O	*Croton draconoides, Camellia sinensis, Maytenus senegalensis*	-7.2	Flavonoid
52.	(-)-Epigallocatechin gallate	C1C(C(OC2=CC(=CC(=C21)O)O)C3=CC(=C(C(=C3)O)O)O)OC(=O)C4=CC(=C(C(=C4)O)O)O	*Camellia sinensis*	-7.7	Flavonoid
53.	Uvaol	CC1CCC2(CCC3(C(=CCC4C3(CCC5C4(CCC(C5(C)C)O)C)C)C2C1C)C)CO	*Crataegus pinnatifida*	-7.9	Terpene
54.	Quercetin 3-O-(2'',6''-digalloyl)-Beta-D-galactopyranoiside	CC1=CC(=CC(=C1C)C)C(=O)OCC2C(C(C(C(O2)OC3=C(OC4=CC(=CC(=C4C3=O)O)O)C5=CC(=C(C=C5)O)O)OC(=O)C6=CC(=C(C(=C6)O)O)O)O)O	*Acer okamotoanum*	-8.0	Flavonoid
55.	Hyperoside	C1=CC(=C(C=C1C2=C(C(=O)C3=C(C=C(C=C3O2)O)O)OC4C(C(C(C(O4)CO)O)O)O)O)O	*Acer okamotoanum*	-8.5	Flavonoid
56.	Quercitrin	CC1C(C(C(C(O1)OC2=C(OC3=CC(=CC(=C3C2=O)O)O)C4=CC(=C(C=C4)O)O)O)O)O	*Acer okamotoanum*	-8.9	Flavonoid
57.	Afzelin	CC1C(C(C(C(O1)OC2=C(OC3=CC(=CC(=C3C2=O)O)O)C4=CC=C(C=C4)O)O)O)O	*Acer okamotoanum*	-8.7	Flavonoid
58.	Kaempferol 3-O-arabinoside	C1C(C(C(C(O1)OC2=C(OC3=CC(=CC(=C3C2=O)O)O)C4=CC=C(C=C4)O)O)O)O	*Acer okamotoanum*	-8.7	Flavonoid
59.	Quercetin 3-O-(2''galloyl)-alpha-L-arabinopyranoside	C1C(C(C(C(O1)OOC2=C(OC3=CC(=CC(=C3C2=O)O)O)C4=CC(=C(C=C4)O)O)OC(=O)C5=CC(=C(C(=C5)O)O)O)O)O	*Acer okamotoanum*	-9.0	Flavonoid
60.	2'-O-Galloylhyperin	C1=CC(=C(C=C1C2=C(C(=O)C3=C(C=C(C=C3O2)O)O)OC4C(C(C(C(O4)CO)O)O)OC(=O)C5=CC(=C(C(=C5)O)O)O)O)O	*Acer okamotoanum*	-8.6	Flavonoid
61.	Methyl gallate	COC(=O)C1=CC(=C(C(=C1)O)O)O	*Acer okamotoanum*	-5.6	Phenolic
62.	1,2,6-Tri-O-galloyl-beta-D-glucose	C1=C(C=C(C(=C1O)O)O)C(=O)OCC2C(C(C(O2)O)(COC(=O)C3=CC(=C(C(=C3)O)O)O)O)OC(=O)C4=CC(=C(C(=C4)O)O)O	*Acer okamotoanum*	-8.5	Tannin
63.	1,2,3,4,6-Penta-O-galloyl-beta-D-glucose	C1=C(C=C(C(=C1O)O)O)C(=O)OCC2C(C(C(C(O2)OC(=O)C3=CC(=C(C(=C3)O)O)O)OC(=O)C4=CC(=C(C(=C4)O)O)O)OC(=O)C5=CC(=C(C(=C5)O)O)O)OC(=O)C6=CC(=C(C(=C6)O)O)O	*Acer okamotoanum*	-8.2	Tannin

64.	3,5-Dicaffeoylquinic acid	C1C(C(C(CC1(C(=O)O)O)OC(=O)C=CC2=CC(=C(C=C2)O)O)O)OC(=O)C=CC3=CC(=C(C=C3)O)O	*Baccharis genistelloides*	-8.3	Phenolic
65.	1-Methoxyoxalyl-3,5-dicaffeoylquinic acid	COC(=O)C(=O)OC1(CC(C(C(C1)OC(=O)C=CC2=CC(=C(C=C2)O)O)O)OC(=O)C=CC3=CC(=C(C=C3)O)O)C(=O)O	*Achyroclinesatureioides*	-7.8	Phenolic
66.	4'-Methyl-epigallocatechin	COC1=C(C=C(C=C1O)C2C(CC3=C(C=C(C=C3O2)O)O)O)O	*Maytenus senegalensis*	-6.8	Phenolic
67.	(-)-4'-Methylepigallocatechin 5-O-.beta.-D-glucopyranoside	COC1=C(C=C(C=C1O)C2C(CC3=C(O2)C=C(C=C3OC4C(C(C(C(O4)CO)O)O)O)O)O)O	*Maytenus senegalensis*	-7.9	Phenolic
68.	(+)-4'-Methylgallocatechin 3'-O-.beta.-D-glucopyranoside	COC1=C(C=C(C=C1OC2C(C(C(C(O2)CO)O)O)O)C3C(CC4=C(C=C(C=C4O3)O)O)O)O	*Maytenus senegalensis*	-8.1	Phenolic
69.	(-)-Epicatechin-(4beta→8)-(-)-epigallocatechin	C1C(C(OC2=C1C(=CC(=C2C3C(C(OC4=CC(=CC(=C34)O)O)C5=CC(=C(C=C5)O)O)O)O)O)C6=CC(=C(C(=C6)O)O)O)O	*Maytenus senegalensis*	-9.3	Phenolic
70.	(-)-Epicatechin(4.beta.→8)(-)-4'-methylepigallocatechin	COC1=C(C=C(C=C1O)C2C(CC3=C(O2)C(=C(C=C3O)O)C4C(C(OC5=CC(=CC(=C45)O)O)C6=CC(=C(C=C6)O)O)O)O)O	*Maytenus senegalensis*	-9.2	Phenolic
71.	Procyanidin B2	C1C(C(OC2=C1C(=CC(=C2C3C(C(OC4=CC(=CC(=C34)O)O)C5=CC(=C(C=C5)O)O)O)O)O)C6=CC(=C(C=C6)O)O)O	*Maytenus senegalensis*	-9.3	Flavonoid
72.	Phlorin	C1=C(C=C(C=C1O)OC2C(C(C(C(O2)CO)O)O)O)O	*Maytenus senegalensis*	-6.3	Phenolic
73.	Vaticinone	CC(CC=CC(=O)C)C1CCC2(C1(CCC34C2CCC5C3(C4)CCC(=O)C5(C)C)C)C	*Vatica cinerea*	-7.5	Terpene
74.	Mangiferonic acid	CC(CCC=C(C)C(=O)O)C1CCC2(C1(CCC34C2CCC5C3(C4)CCC(=O)C5(C)C)C)C	*Vatica cinerea*	-7.8	Terpene
75.	Dihydroschizandronic acid	CC(CCCC(C)C(=O)O)C1CCC2(C1(CCC34C2CCC5C3(C4)CCC(=O)C5(C)C)C)C	*Vatica cinerea*	-7.3	Terpene
76.	Anwuweizonic acid	CC(CCC=C(C)C(=O)O)C1CCC2(C1(CCC3=C2CCC4C3(CCC(=O)C4(C)C)C)C)C	*Vatica cinerea*	-7.4	Terpene
77.	(24S)-Dammara-20,25-diene-3beta,24-diol	CC(=C)C(CCC(=C)C1CCC2(C1CCC3C2(CCC4C3(CCC(C4(C)C)O)C)C)C)O	*Vatica cinerea*	-7.9	Terpene
78.	(17alpha,23E)-5alpha-Dammara-20,23-diene-3beta,25-diol	CC1(C2CCC3(C(C2(CCC1O)C)CCC4C3(CCC4C(=C)CC=CC(C)(C)O)C)C)C	*Vatica cinerea*	-7.8	Terpene
79.	Betulin	CC(=C)C1CCC2(C1C3CCC4C5(CCC(C(C5CCC4(C3(CC2)C)C)(C)C)O)C)CO	*Vatica cinerea*	-7.3	Terpene

80.	Betulonic acid	CC(=C)C1CCC2(C1C3CCC4C5(CCC(=O)C(C5CCC4(C3(CC2)C)C)(C)C)C)C(=O)O	*Vatica cinerea*	-7.9	Terpene
81.	Erythrodiol	CC1(CCC2(CCC3(C(=CCC4C3(CCC5C4(CCC(C5(C)C)O)C)C)C2C1)C)CO)C	*Vatica cinerea*	-8.0	Terpene
82.	Pheophorbide A	CCC1=C(C2=NC1=CC3=C(C4=C(C(C(=C5C(C(C(=CC6=NC(=C2)C(=C6C)C=C)N5)C)CCC(=O)O)C4=N3)C(=O)OC)O)C)C	*Vatica cinerea*	-8.3	Terpene
83.	1-Hydroxycyclocolorenone	CC1CCC2C(C2(C)C)C3=C(C(=O)CC13O)C	*Vatica cinerea*	-6.6	Terpene
84.	Platanic acid	CC(=O)C1CCC2(C1C3CCC4C5(CCC(C(C5CCC4(C3(CC2)C)C)(C)C)O)C)C(=O)O	*Syzygiumclaviflorum*	-8.1	Terpene
85.	Andrographolide	CC12CCC(C(C1CCC(=C)C2CC=C3C(COC3=O)O)(C)CO)O	*Andrographis paniculata*	-6.7	Terpene
86.	1-Methoxycanthinone	COC1=CN=C2C=CC(=O)N3C2=C1C4=CC=CC=C43	*Leitneriafloridana*	-7.2	Alkaloid
87.	Ovatodiolide	CC1=CCCC2=CC(CC(=CC3C(CC1)C(=C)C(=O)O3)C)OC2=O	*Anisomeles indica*	-7.7	Terpene
88.	Panduratin A	CC1=CCC(C(C1CC=C(C)C)C(=O)C2=C(C=C(C=C2O)OC)O)C3=CC=CC=C3	*Boesenbergiapandurata*	-6.6	Flavonoid
89.	Panduratin C	CC1=CCC(C(C1CC=C(C)C)C(=O)C2=C(C=C(C=C2OC)O)O)C3=CC=C(C=C3)O	*Boesenbergiapandurata*	-7.1	Flavonoid
90.	4-Hydroxypanduratin A	CC1=CCC(C(C1CC=C(C)C)C(=O)C2=C(C=C(C=C2O)O)O)C3=CC=CC=C3	*Boesenbergiapandurata*	-6.8	Flavonoid
91.	Helichrysetin	COC1=CC(=CC(=C1C(=O)C=CC2=CC=C(C=C2)O)O)O	*Boesenbergiapandurata*	-7.1	Flavonoid
92.	Uvangoletin	COC1=CC(=CC(=C1C(=O)CCC2=CC=CC=C2)O)O	*Boesenbergiapandurata*	-6.5	Flavonoid
93.	Drymaritin	COC1=CC(=O)N2C3=CC=CC=C3C4=C2C1=NC=C4	*Drymariadiandra*	-7.0	Alkaloid
94.	Calophyllolide	CC=C(C)C(=O)C1=C(C2=C(C3=C1OC(=O)C=C3C4=CC=CC=C4)OC(C=C2)(C)C)OC	*Calophylluminophyllum*	-7.7	Coumarin
95.	Inophyllum B	CC1C(OC2=C(C1O)C3=C(C(=CC(=O)O3)C4=CC=CC=C4)C5=C2C=CC(O5)(C)C)C	*Calophylluminophyllum*	-8.3	Coumarin
96.	Inophyllum C	CC1C(OC2=C(C1=O)C3=C(C(=CC(=O)O3)C4=CC=CC=C4)C5=C2C=CC(O5)(C)C)C	*Calophylluminophyllum*	-8.6	Coumarin
97.	Inophyllum G-1	CC1C(OC2=C(C1O)C3=C(C(=CC(=O)O3)C4=CC=CC=C4)C5=C2C6C(C6(C)C)O5)C	*Calophylluminophyllum*	-8.5	Coumarin
98.	Inophyllum G-2	CC1C(OC2=C(C1O)C3=C(C(=CC(=O)O3)C4=CC=CC=C4)C5=C2C6C(C6(C)C)O5)C	*Calophylluminophyllum*	-8.7	Coumarin
99.	Inophyllum P	CC1C(OC2=C(C1O)C3=C(C(=CC(=O)O3)C4=CC=CC=C4)C5=C2C=CC(O5)(C)C)C	*Calophylluminophyllum*	-8.3	Coumarin

100.	1-Deoxyforskolin	CC(=O)OC1C(C2C(CCCC2(C3(C1(OC(CC3=O)(C)C=C)C)O)C)(C)C)O	*Coleus forskohlii*	-6.7	Terpene
101.	1,9-Dideoxyforskolin	CC(=O)OC1C(C2C(CCCC2(C3C1(OC(CC3=O)(C)C=C)C)C)(C)C)O	*Coleus forskohlii*	-6.5	Terpene
102.	Forskolin	CC(=O)OC1C(C2C(CCC(C2(C3(C1(OC(CC3=O)(C)C=C)C)O)C)O)(C)C)O	*Coleus forskohlii*	-6.4	Terpene
103.	Isoforskolin	CC(=O)OC1C2C(CCC(C2(C3(C(=O)CC(OC3(C1O)C)(C)C=C)O)C)O)(C)C	*Coleus forskohlii*	-6.5	Terpene
104.	Scutellarin	C1=CC(=CC=C1C2=CC(=O)C3=C(C(=C(C=C3O2)OC4C(C(C(C(O4)C(=O)O)O)O)O)O)O)O	*Erigeron breviscapus*	-8.2	Flavonoid
105.	8-Prenylluteone	CC(=CCC1=C(C(=C2C(=C1O)C(=O)C(=CO2)C3=C(C=C(C=C3)O)O)CC=C(C)C)O)C	*Erythrina senegalensis*	-7.8	Flavonoid
106.	Auriculatin	CC(=CCC1=C2C(=C(C3=C1OC=C(C3=O)C4=C(C=C(C=C4)O)O)O)C=CC(O2)(C)C)C	*Erythrina senegalensis*	-7.9	Flavonoid
107.	Erysenegalensein O	CC(=CCC1=C2C(=C3C(=C1O)C(=O)C(=CO3)C4=C(C=C(C=C4)O)O)CC(C(O2)(C)C)O)C	*Erythrina senegalensis*	-8.1	Flavonoid
108.	Erysenegalensein D	CC(=CCC1=C(C(=C2C(=C1O)C(=O)C(=CO2)C3=C(C=C(C=C3)O)O)CC(C(=C)C)O)O)C	*Erythrina senegalensis*	-7.9	Flavonoid
109.	Erysenegalensein N	CC(=CCC1=C(C(=C(C2=C1OC=C(C2=O)C3=C(C=C(C=C3)O)O)O)CC(C(=C)C)O)O)C	*Erythrina senegalensis*	-7.4	Flavonoid
110.	Derrone	CC1(C=CC2=C(O1)C=C(C3=C2OC=C(C3=O)C4=CC=C(C=C4)O)O)C	*Erythrina senegalensis*	-8.0	Flavonoid
111.	Alpinumisoflavone	CC1(C=CC2=C(O1)C=C3C(=C2O)C(=O)C(=CO3)C4=CC=C(C=C4)O)C	*Erythrina senegalensis*	-7.5	Flavonoid
112.	6,8-Diprenylgenistein	CC(=CCC1=C(C(=C2C(=C1O)C(=O)C(=CO2)C3=CC=C(C=C3)O)CC=C(C)C)O)C	*Erythrina senegalensis*	-7.9	Flavonoid
113.	2-Isoprenylforbesione	CC(=CCC1=C(C(=C2C(=C1O)C(=O)C3=CC4CC5C3(O2)C(C4=O)(OC5(C)C)CC=C(C)C)CC=C(C)C)O)C	*Garcinia hanburyi*	-7.5	Xanthone
114.	8,8a-Epoxymorellic acid	CC(=CCC1=C2C(=C(C3=C1OC45C6C(=O)C(CC4(C(O6)(C)C)CC=C(C)C(=O)O)C7C5(C3=O)O7)O)C=CC(O2)(C)C)C	*Garcinia hanburyi*	-8.1	Xanthone
115.	Desoxymorellin	CC(=CCC1=C2C(=C(C3=C1OC45C6CC(C=C4C3=O)C(=O)C5(OC6(C)C)CC=C(C)C)O)C=CC(O2)(C)C)C	*Garcinia hanburyi*	-8.2	Xanthone
116.	Isomorellin	CC(=CCC1=C2C(=C(C3=C1OC45C6CC(C=C4C3=O)C(=O)C5(OC6(C)C)CC=C(C)C=O)O)C=CC(O2)(C)C)C	*Garcinia hanburyi*	-7.5	Xanthone
117.	Isomorellinol	CC(=CCC1=C2C(=C(C3=C1OC45C6CC(C=C4C3=O)C(=O)C5(OC6(C)C)CC=C(C)CO)O)C=CC(O2)(C)C)C	*Garcinia hanburyi*	-8.1	Xanthone

118.	Morellic acid	CC(=CCC1=C2C(=C(C3=C1OC45C6CC(C=C4C3=O)C(=O)C5(OC6(C)C)CC=C(C)C(=O)O)O)C=CC(O2)(C)C)C	*Garcinia hanburyi*	-8.3	Xanthone
119.	Gambogic acid	CC(=CCCC1(C=CC2=C(C3=C(C(=C2O1)CC=C(C)C)OC45C6CC(C=C4C3=O)C(=O)C5(OC6(C)C)CC=C(C)C(=O)O)O)C)C	*Garcinia hanburyi*	-8.7	Xanthone
120.	Desoxygambogenin	CC(=CCCC(=CCC1=C(C(=C2C(=C1O)C(=O)C3=CC4CC5C3(O2)C(C4=O)(OC5(C)C)CC=C(C)C)CC=C(C)C)O)C)C	*Garcinia hanburyi*	-7.8	Xanthone
121.	Forbesione	CC(=CCC1=C2C(=C(C=C1O)O)C(=O)C3=CC4CC5C3(O2)C(C4=O)(OC5(C)C)CC=C(C)C)C	*Garcinia hanburyi*	-7.7	Xanthone
122.	Dihydroisomorellin	CC(=CCC1=C2C(=C(C3=C1OC45C(C3=O)CC6CC4C(OC5(C6=O)CC=C(C)C=O)(C)C)O)C=CC(O2)(C)C)C	*Garcinia hanburyi*	-8.6	Xanthone
123.	Wikstrol B	C1C(C(OC2=C1C(=CC(=C2C3=C(OC4=CC(=CC(=C4C3=O)O)O)C5=CC=C(C=C5)O)O)O)C6=CC=C(C=C6)O)O	*Wikstroemia indica*	-9.0	Flavonoid
124.	Ginkgolic acid	CCCCCCC=CCCCCCCCC1=C(C(=CC=C1)O)C(=O)O	*Ginkgo biloba*	-5.7	Phenolic
125.	Ginkgolide A	CC1C(=O)OC2C1(C34C(=O)OC5C3(C2)C6(C(C5)C(C)(C)C)C(C(=O)OC6O4)O)O	*Ginkgo biloba*	-7.7	Terpene
126.	Ginkgolide B	CC1C(=O)OC2C1(C34C(=O)OC5C3(C2O)C6(C(C5)C(C)(C)C)C(C(=O)OC6O4)O)O	*Ginkgo biloba*	-7.9	Terpene
127.	3-Hydroxy lauric acid	CCCCCCCCCC(CC(=O)O)O	*Hypericum perforatum*	-4.9	Fatty acid
128.	Patentiflorin A	CC1C(C(C(C(O1)OC2=C3COC(=O)C3=C(C4=CC(=C(C=C42)OC)OC)C5=CC6=C(C=C5)OCO6)O)O)O	*Justicia gendarussa*	-8.0	Lignin
129.	12-Acetoxycalanolide A	CCCC1=CC(=O)OC2=C1C3=C(C=CC(O3)(C)C)C4=C2C(C(C(O4)C)C)OC(=O)C	*Calophyllumlanigerum*	-7.5	Coumarin
130.	12-Methoxycalanolide A	CCCC1=CC(=O)OC2=C1C3=C(C=CC(O3)(C)C)C4=C2C(C(C(O4)C)C)OC	*Calophyllumlanigerum*	-7.3	Coumarin
131.	12-Methoxycalanolide B	CCCC1=CC(=O)OC2=C1C3=C(C=CC(O3)(C)C)C4=C2C(C(C(O4)C)C)OC	*Calophyllumlanigerum*	-7.2	Coumarin
132.	Calanolide B	CCCC1=CC(=O)OC2=C1C3=C(C=CC(O3)(C)C)C4=C2C(C(C(O4)C)C)O	*Calophyllumlanigerum*	-7.6	Coumarin
133.	Calanolide C	CCCC1=CC(=O)OC2=C1C3=C(C=CC(O3)(C)C)C4=C2C(C(C(O4)C)C)O	*Calophyllumlanigerum*	-7.6	Coumarin
134.	Scirpusin A	C1=CC(=CC=C1C=CC2=C3C(C(OC3=CC(=C2)O)C4=CC(=C(C=C4)O)O)C5=CC(=CC(=C5)O)O)O	*Caragana rosea*	-8.6	Stilbene
135.	Scirpusin B	C1=CC(=C(C=C1C=CC2=C3C(C(OC3=CC(=C2)O)C4=CC(=C(C=C4)O)O)C5=CC(=CC(=C5)O)O)O)O	*Caragana rosea*	-8.8	Stilbene

136.	Salaspermic acid	CC1C23CCC4C(C2CCC1(OC3)O)(CCC5(C4(CCC6(C5CC(CC6)(C)C(=O)O)C)C)C)C	*Tripterygium wilfordii*	-8.6	Terpene
137.	Suberosol	CC(C)C(=C)CCC(C)C1CC(C2(C1(CC=C3C2=CCC4C3(CCC(C4(C)C)O)C)C)C)O	*Polyalthiasuberosa*	-7.1	Terpene
138.	Cucurbitacin F	CC1(C(C(CC2C1=CCC3C2(C(=O)CC4(C3(CC(C4C(C)(C(=O)C=CC(C)(C)O)O)O)C)C)C)O)O)C	*Cowania mexicana*	-7.4	Terpene
139.	Suksdorfin	CC(C)CC(=O)OC1C(C(OC2=C1C3=C(C=C2)C=CC(=O)O3)(C)C)OC(=O)C	*Lomatium suksdorfii*	-7.1	Coumarin
140.	Baicalin	C1=CC=C(C=C1)C2=CC(=O)C3=C(C(=C(C=C3O2)OC4C(C(C(C(O4)C(=O)O)O)O)O)O)O	*Scutellaria baicalensis*	-8.3	Flavonoid
141.	Norisoboldine	COC1=C(C2=C3C(CC4=CC(=C(C=C42)OC)O)NCCC3=C1)O	*Croton echinocarpus*	-7.7	Alkaloid
142.	Corydine	CN1CCC2=CC(=C(C3=C2C1CC4=C3C(=C(C=C4)OC)OC)O)OC	*Croton echinocarpus*	-7.0	Alkaloid
143.	Actein	CC1CC2(C3C(O3)(C(O2)O)C)OC4C1C5(C(CC67CC68CCC(C(C8CCC7C5(C4)C)(C)C)OC9C(C(C(CO9)O)O)O)OC(=O)C)C	*Cimicifuga racemosa*	-8.9	Terpene
144.	Nigranoic acid	CC(CCC=C(C)C(=O)O)C1CCC2(C1(CCC34C2CCC(C3(C4)CCC(=O)O)C(=C)C)C)C	*Schisandra sphaerandra*	-7.1	Terpene
145.	Corilagin	C1C2C(C(C(C(O2)OC(=O)C3=CC(=C(C(=C3)O)O)O)O)OC(=O)C4=CC(=C(C(=C4C5=C(C(=C(C=C5C(=O)O1)O)O)O)O)O)O)O	*Chamaesyce hyssopifolia*	-8.7	Phenolic
146.	Anolignan B	C=C(CC1=CC=C(C=C1)O)C(=C)CC2=CC=C(C=C2)O	*Anogeissus acuminata*	-5.7	Lignan
147.	Macluraxanthone B	CC(=CCC1=C(C(=C(C2=C1OC3=CC(=C(C=C3C2=O)O)O)O)O)C(C)(C)C=C)O)C	*Maclura tinctoria*	-8.0	Xanthone
148.	Limonin	CC1(C2CC(=O)C3(C(C24COC(=O)CC4O1)CCC5(C36C(O6)C(=O)OC5C7=COC=C7)C)C)C	*Citrus* spp.	-8.7	Terpene
149.	Nomilin	CC(=O)OC1CC(=O)OC(C2C1(C3CCC4(C(OC(=O)C5C4(C3(C(=O)C2)C)O5)C6=COC=C6)C)C)(C)C	*Citrus* spp.	-8.1	Terpene
150.	Lancilactone C	CC1=CCC(OC1=O)C(C)C2CCC3(C2(CCC4=C3C=CC(=C(C)C)C(=C4)CCC(=O)O)C)C	*Kadsura lanci-limba*	-8.8	Terpene

Table S2. Docking results of selected drugs and inhibitor against M^pro^ of SARS-CoV-2 (PDB: 6LU7)

Sl. No.	Name of Compound	Canonical SMILES	Binding energy (kcal/mol)
1.	Atazanavir	CC(C)(C)C(C(=O)NC(CC1=CC=CC=C1)C(CN(CC2=CC=C(C=C2)C3=CC=CC=N3)NC(=O)C(C(C)(C)C)NC(=O)OC)O)NC(=O)OC	-6.9
2.	Darunavir	CC(C)CN(CC(C(CC1=CC=CC=C1)NC(=O)OC2COC3C2CCO3)O)S(=O)(=O)C4=CC=C(C=C4)N	-7.7
3.	Lopinavir	CC1=C(C(=CC=C1)C)OCC(=O)NC(CC2=CC=CC=C2)C(CC(CC3=CC=CC=C3)NC(=O)C(C(C)C)N4CCCNC4=O)O	-7.3
4.	Maraviroc	CC1=NN=C(N1C2CC3CCC(C2)N3CCC(C4=CC=CC=C4)NC(=O)C5CCC(CC5)(F)F)C(C)C	-8.7
5.	Cenicriviroc	CCCCOCCOC1=CC=C(C=C1)C2=CC3=C(C=C2)N(CCCC(=C3)C(=O)NC4=CC=C(C=C4)S(=O)CC5=CN=CN5CCC)CC(C)C	-7.7
6	Efavirenz	C1CC1C#CC2(C3=C(C=CC(=C3)Cl)NC(=O)O2)C(F)(F)F	-7.1
7.	Nevirapine	CC1=C2C(=NC=C1)N(C3=C(C=CC=N3)C(=O)N2)C4CC4	-6.6
8.	Rilpivirine	CC1=CC(=CC(=C1NC2=NC(=NC=C2)NC3=CC=C(C=C3)C#N)C)C=CC#N	-8.9
9.	Raltegravir	CC1=NN=C(O1)C(=O)NC(C)(C)C2=NC(=C(C(=O)N2C)O)C(=O)NCC3=CC=C(C=C3)F	-8.1
10.	Elvitegravir	CC(C)C(CO)N1C=C(C(=O)C2=C1C=C(C(=C2)CC3=C(C(=CC=C3)Cl)F)OC)C(=O)O	-7.5
11.	Dolutegravir	CC1CCOC2N1C(=O)C3=C(C(=O)C(=CN3C2)C(=O)NCC4=C(C=C(C=C4)F)F)O	-8.7
12.	Abacavir	C1CC1NC2=C3C(=NC(=N2)N)N(C=N3)C4CC(C=C4)CO	-7.2
13.	Emtricitabine	C1C(OC(S1)CO)N2C=C(C(=NC2=O)N)F	-6.3
14.	Lamivudine	C1C(OC(S1)CO)N2C=CC(=NC2=O)N	-6.0
15.	Tenofovir	CC(CN1C=NC2=C(N=CN=C21)N)OCP(=O)(O)O	-6.4
16.	Zidovudine	CC1=CN(C(=O)NC1=O)C2CC(C(O2)CO)N=[N+]=[N-]	-7.1
17.	Remdesivir	CCC(CC)COC(=O)C(C)NP(=O)(OCC1C(C(C(O1)(C#N)C2=CC=C3N2N=CN=C3N)O)O)OC4=CC=CC=C4	-7.8
18.	Tipranavir	CCCC1(CC(=C(C(=O)O1)C(CC)C2=CC(=CC=C2)NS(=O)(=O)C3=NC=C(C=C3)C(F)(F)F)O)CCC4=CC=CC=C4	-8.4
19.	Inhibitor N3	CC1=CC(=NO1)C(=O)NC(C)C(=O)NC(C(C)C)C(=O)NC(CC(C)C)C(=O)NC(CC2CCNC2=O)C=CC(=O)OCC3=CC=CC=C3	-7.9

Table S3. Lipophilicity and Pharmacokinetics of the Selected Potential Biomolecules

**Sl. No.**	**1**	**2**	**3**	**4**	**5**	**6**	**7**	**8**	**9**	**10**	**11**	**12**	**13**	**14**	**15**	**16**	**17**	**N3**
**Lipophilicity**																		
iLOGP	2.94	3.10	3.06	2.05	1.43	2.18	3.02	3.53	5.70	2.77	2.17	1.93	2.24	1.99	1.27	4.07	3.23	3.99
XLOGP3	4.46	5.71	5.04	2.37	2.01	2.34	5.04	5.65	8.32	4.49	2.75	1.64	4.15	4.47	0.86	3.33	4.55	3.35
WLOGP	4.73	5.76	5.13	2.35	2.05	2.35	5.13	5.55	7.64	4.35	1.55	1.01	4.07	4.18	0.49	3.03	4.88	1.55
MLOGP	0.58	1.36	0.25	-0.26	-0.74	-0.55	0.25	0.52	3.18	0.58	-1.65	-2.15	0.48	-0.08	-1.84	1.63	2.37	0.38
SILICOS-IT	4.77	5.37	4.61	1.14	0.67	1.22	4.61	4.63	8.27	3.68	1.13	-0.45	3.56	3.25	0.01	2.46	4.04	4.42
Consensus Log $P_{o/w}$	3.50	4.26	3.62	1.53	1.08	1.51	3.61	3.98	6.62	3.17	1.19	0.39	2.90	2.76	0.16	2.90	3.82	2.74
**Pharmacokinetics**																		
GIabsorption	Low	Low	Low	Low	Low	Low	Low	Low	Low	Low	Low	Low	Low	Low	Low	Low	High	Low
BBBpermeation	No	No	No	No	No	No	No	No	No	No	No	No	No	No	No	No	No	No
P-gpsubstrate	No	No	No	No	No	No	No	No	No	No	No	No	No	No	No	Yes	No	Yes
CYP1A2inhibitor	No	No	No	No	No	No	No	No	No	No	No	No	No	No	No	No	Yes	No
CYP2C19inhibitor	Yes	Yes	No	No	No	No	No	No	No	No	No	No	No	No	No	No	Yes	No
CYP2C9inhibitor	Yes	Yes	No	No	No	No	No	Yes	No	Yes	No	No	Yes	Yes	No	No	Yes	No
CYP2D6inhibitor	No	No	No	No	No	No	No	No	No	No	No	No	No	No	No	No	No	No
LogKp(skin permeation)(cm/s)	-6.31	-5.32	-6.01	-8.15	-8.50	-8.35	-6.01	-5.57	-5.01	-6.42	-8.65	-8.81	-6.66	-6.52	-8.42	-8.06	-5.30	-8.07

Table S4. Drug likeness of the Selected Potential Biomolecules

Sl. No.	Lipinski (Pfizer)	Ghose	Veber (GSK)	Egan (Pharmacia)	Muegge (Bayer)	Bioavailability Score
1	No; 2 violations<br>MW>500<br>NH or OH >5	No; 2 violations<br>MW>480, MR>130	No; 1 violation<br>TPSA>140	No; 1 violation<br>TPSA>131.6	No; 3 violations<br>TPSA>150, #rings >7<br>H-don>5	0.17
2	No; 2 violations<br>MW>500<br>NH or OH >5	No; 3 violations<br>MW>480,MR>130<br>WLOGP >5.6	No; 1 violation<br>TPSA>140	No; 1 violation<br>TPSA>131.6	No; 4 violations<br>TPSA>150,#rings>7<br>H-don >5, XLOGP >3.5	0.17
3	No; 2 violations<br>MW >500<br>NH or OH >5	No; 3 violations<br>MW >480, MR >130	No; 1 violation<br>TPSA >140	No; 1 violation<br>TPSA >131.6	No; 3 violations<br>TPSA >150, XLOGP >3.5<br>H-don >5	0.17
4	No; 3 violations<br>MW >500, N or O >10<br>NH or OH >5	No; 3violations<br>MW >480, MR >130	No; 1violation<br>TPSA >140	No; 1violation<br>TPSA >131.6	No; 3violations<br>TPSA >150, H-acc >10<br>H-don>5	0.17
5	No; 3 violations<br>MW >500, N or O >10<br>NH or OH >5	No; 3 violations<br>MW >480, MR >130	No; 1 violation<br>TPSA >140	No; 1 violation<br>TPSA >131.6	No; 3 violations<br>TPSA >150,H-acc >10<br>H-don>5	0.17
6	No; 3 violations<br>MW >500, N or O >10<br>NH or OH >5	No; 3 violations<br>MW >480, MR >130<br>#atoms >70	No; 1 violation<br>TPSA >140	No; 1 violation<br>TPSA >131.6	No; 4 violations<br>TPSA >150, H-acc >10<br>H-don >5, MW >600	0.17
7	No; 2 violations<br>MW >500<br>NH or OH >5	No; 2 violations<br>MW >480, MR >130	No; 1 violation<br>TPSA >140	No; 1 violation<br>TPSA >131.6	No; 3 violations<br>TPSA >150, XLOGP >3.5<br>H-don>5	0.17
8	Yes; 1 violation<br>MW >500	No; 2 violations<br>MW >480, MR>130	No; 1 violation<br>TPSA >140	No; 1 violation<br>TPSA >131.6	No; 2 violations<br>TPSA >150, XLOGP >3.5	0.55
9	No; 2 violations<br>MW >500<br>NH or OH >5	No; 4 violations<br>MW >480, MR >130<br>WLOGP >5.6<br>#atoms >70	No; 1 violation<br>TPSA >140	No; 2 violations<br>TPSA >131.6<br>WLOGP >5.88	No; 5 violations<br>TPSA >150, #rings >7<br>H-don >5, XLOGP >3.5<br>MW >600	0.17
	No; 2 violations	No; 2 violations	No; 1 violation	No; 1 violations	No; 2 violations	

10	MW >500	MW >480, MR >130	TPSA >140	TPSA >131.6	TPSA >150, H-don >5	0.17
	NH or OH >5					
	No; 3 violations	No; 3 violations	No; 1 violation	No; 1 violations	No; 4 violations	
11	MW >500, NorO>10	MW >480, MR >130	TPSA >140	TPSA >131.6	TPSA >150, H-don >5	0.17
	NH or OH >5	#atoms >70			MW >600, H-don >5	
	No; 3 violations	No; 2 violations	No; 1 violation	No; 1 violations	No; 4 violations	
12	MW >500, NorO>10	MW >480, MR >130	TPSA >140	TPSA >131.6	TPSA >150, H-don >5	0.17
	NH or OH >5				MW>600,H-acc>10	
	No; 2 violations	No; 2 violations	No; 1 violation	No; 1 violations	No; 2 violations	
13	MW >500	MW >480, MR >130	TPSA >140	TPSA >131.6	TPSA >150, H-don >5	0.17
	NH or OH>5					
	No; 3 violations	No; 2 violations	No; 1 violation	No; 1 violations	No; 3 violations	
14	MW >500,N or O >10	MW >480, MR >130	TPSA >140	TPSA >131.6	TPSA >150, H-don >5	0.17
	NH or OH >5				H-acc>0	
	No; 2 violations		No; 1 violation	No; 1 violations	No; 3 violations	
15	N or O >10	Yes	TPSA >140	TPSA >131.6	TPSA >150, H-don >5	0.17
	NH or OH>5				H-acc >10	
	No; 2 violations	No; 3 violations	No; 1 violation	No; 1 violations	No; 4 violations	
16	N or O>10	MW >480, MR >130	TPSA >140	TPSA >131.6	TPSA >150, MW >600	0.17
	MW >500	#atoms >70			H-acc >10, #rings >7	
17	Yes; 0 violation	Yes	Yes	Yes	Yes	0.55
	No; 2 violations	No; 3 violations	No; 2 violations	No; 1 violation	No; 3 violations	
N3	MW >500	MW >480, MR >130	Rotors >10	TPSA >131.6	MW >600, TPSA >150	0.17
	N or O >10	#atoms >70	TPSA >140		Rotors >15	

Table S5. Medicinal Chemistry Friendliness of the Selected Potential Biomolecules

Sl. No.	PAINS	Brenk	Lead likeness	Synthetic Accessibility Score
1	1 alert; ene_one_D	2 alerts: polycyclic_aromatic_hydrocarbon_2 polycyclic_aromatic_hydrocarbon_3	No; 2 violations: MW >350, XLOGP3 >3.5	3.95
2	1 alert; ene_one_D	2 alerts:polycyclic_aromatic_hydrocarbon_2 polycyclic_aromatic_hydrocarbon_3	No; 2 violations: MW >350, XLOGP3 >3.5	3.89
3	0 alert	0 alert	No; 2 violations: MW >350, XLOGP3 >3.5	4.19
4	1 alert: catechol.A	1 alert: catechol.A	No; 1 violation: MW>350	5.32
5	1 alert: catechol.A	1 alert: catechol.A	No; 1 violation: MW>350	5.36
6	1 alert: satechol.A	1 alert: catechol.A	No; 1 violation: MW>350	5.49
7	0 alert	0 alert	No; 2 violations: MW >350, XLOGP3 >3.5	4.17
8	0 alert	0 alert	No; 2 violations: MW >350, XLOGP3>3.5	4.05
9	1 alert: mannich, A	0 alert	No; 2 violations: MW>350, XLOGP3 >3.5	6.29
10	0 alert	0 alert	No; 2 violations: MW>350, XLOGP3 >3.5	4.66
11	0 alert	0 alert	No; 1 violation: MW >350	6.56
12	1 alert: satechol.A.	2 alerts: catechol, peroxide	No; 1 violation: MW >350	5.95
13	0 alert	0 alert	No; 2 violations: MW >350, XLOGP3 >3.5	5.09
14	1 alert: catechol.A.	1 alert: catechol	No; 2 violations: MW >350, XLOGP3 >3.5	5.03
15	1 alert:	1 alert: catechol	No; 1 violation: MW >350	5.28
16	0 alert	2 saponine, derivative	No; 1 violation: MW >350	8.77
17	0 alert	1 alert: coniugatednitole group	No; 2 violations: MW>350, XLOGP3 >3.5	3.29
N3	0 alert	1 alert: michael_acceptor_ 1	No: 2 violations: MW >350, Rotors >7	6.43
